# Chemical Analysis of a “Miller-Type” Complex Prebiotic Broth: Part II: Gas, Oil, Water and the Oil/Water-Interface

**DOI:** 10.1007/s11084-016-9528-8

**Published:** 2016-11-28

**Authors:** Sabrina Scherer, Eva Wollrab, Luca Codutti, Teresa Carlomagno, Stefan Gomes da Costa, Andreas Volkmer, Amela Bronja, Oliver J. Schmitz, Albrecht Ott

**Affiliations:** 1 Biologische Experimentalphysik, Universität des Saarlandes, Campus, Geb. B2 1, 66123 Saarbrücken, Germany; 2 Present Address: Laboratory of Microbial Morphogenesis and Growth, Institut Pasteur, 75724 Paris Cedex 15, France; 3 Centre of Biomolecular Drug Research, Leibniz University, Schneiderberg 38, 30167 Hannover, Germany; 4 Coherent Raman Scattering Microscopy and Single-Molecule Spectroscopy Group, 3. Institute of Physics, University of Stuttgart, Pfaffenwaldring 57, 70569 Stuttgart, Germany; 5 Applied Analytical Chemistry, University of Duisburg-Essen, Campus Essen, S05 T01 B35, Universitaetsstr. 5, 45141 Essen, Germany

**Keywords:** Origin of Life, Complex chemical mixture, Miller-Urey experiment, NMR, Molecular vibrations, coherent anti-Stokes Raman scattering (CARS), GC/MS, GCxGC/MS, Oil/water interface, Phase-transfer-catalysis, Radicals

## Abstract

We have analyzed the chemical variety obtained by Miller-Urey-type experiments using nuclear magnetic resonance (NMR) spectroscopy and coherent anti-Stokes Raman scattering (CARS) spectroscopy, gas chromatography followed by mass spectrometry (GC/MS) and two-dimensional gas chromatography followed by mass spectrometry (GCxGC/MS). In the course of a running Miller-Urey-type experiment, a hydrophobic organic layer emerged besides the hydrophilic aqueous phase and the gaseous phase that were initially present. The gas phase mainly consisted of aromatic compounds and molecules containing *C*≡*C* or *C*≡*N* triple bonds. The hydrophilic phase contained at least a few thousands of different molecules, primarily distributed in a range of *50* and *500 Da*. The hydrophobic phase is characterized by carbon-rich, oil-like compounds and their amphiphilic derivatives containing oxygen with tensioactive properties. The presence of a wide range of oxidized molecules hints to the availability of oxygen radicals. We suggest that they intervene in the formation of alkylated polyethylene glycol (PEG) in the oil/water interface. CARS spectroscopy revealed distinct vibrational molecular signatures. In particular, characteristic spectral bands for cyanide compounds were observed if the broth was prepared with electric discharges in the gaseous phase. The characteristic spectral bands were absent if discharges were released onto the water surface. NMR spectroscopy on the same set of samples independently confirmed the observation. In addition, NMR spectroscopy revealed overall high chemical variability that suggests strong non-linearities due to interdependent, sequential reaction steps.

**Electronic supplementary material:**

The online version of this article (doi:10.1007/s11084-016-9528-8) contains supplementary material, which is available to authorized users.

## Introduction

In 1953, Miller and Urey synthesized organic compounds, including amino acids, from water, methane, ammonia, and hydrogen. This was a spectacular experiment that emulated the conditions on the early Earth (Miller 1953). Since then, many experiments related to the origin of life were performed under various conditions (Miyakawa et al. 2002; Oró 1963; Schlesinger and Miller 1983; Fox 1995; Johnson et al. 2008). Besides amino acids, the formation of relevant precursors of biomolecules such as carboxylic acids, urea, and lipids was observed (Dickerson 1979; Dose and Rauchfuss 1975; Lazcano and Bada 2003; McCollom et al. 1999). Here, we describe the chemical analysis of complex prebiotic broth in experiments of the Miller-Urey type. We used nuclear magnetic resonance (NMR) spectroscopy, coherent anti-Stokes Raman scattering (CARS) spectroscopy, gas chromatography/mass spectrometry (GC/MS), and two-dimensional gas chromatography/mass spectrometry (GCxGC/MS). Different analytical methods were necessary to achieve a comprehensive picture of the complex reaction mixture.

Non-invasive, chemical analysis techniques that are based on optical spectroscopy commonly rely on probing the vibrational response of the molecular sample of interest. For the prebiotic broth in this work, we found conventional spontaneous Raman scattering spectroscopy impossible to perform because of the presence of strong and broad auto-fluorescence backgrounds, which masked the observation of any vibrational signature bands. To circumvent this problem, next we used CARS spectroscopy (Volkmer 2005) where detection occurs at spectral frequencies on the anti-Stokes side of the excitation frequencies. Here, no one-photon induced fluorescence background is detected. Moreover, the coherent driving and probing of induced molecular vibrations yield an enhancement of the Raman detection sensitivity by several orders of magnitude. The analysis of a CARS spectrum gives information about vibrational properties of all molecules inside the liquid sample probe volume, typically about one femtoliter.

We used NMR spectroscopy to investigate the degree of complexity of the studied molecular mixtures. NMR spectroscopy not only enables to determine functional groups of the substances under study but also provides information about chemical structures of molecules and their molecular weights. The signal intensities directly correlate with the concentration of the substances and even small molecules (<*100 Da*) are detectable. Unlike CARS spectroscopy, NMR requires a high sample volume of the order of a milliliter.

During each Miller-Urey-type experiment, a very thin hydrophobic layer emerged on top of the water-based broth. It was not possible to withdraw the oil-like phase separately from the aqueous phase. We isolated the hydrophobic substances by extraction with an organic solvent. We analyzed the low concentrated samples using GC/MS and GCxGC/MS that are suitable for the analysis of hydrophobic substances and require only a small sample volume in the microliter range. The two-dimensionality of GCxGC/MS allows for a very high resolution in separating substances, which produce overlapping peaks in GC/MS. The columns used in both techniques exhibit little sensitivity to nitrogen containing compounds.

In (Wollrab et al. 2015), we previously described two types of polymer as part of a Miller-type broth, one based on a nitrogen-carbon, the other on an oxygen-carbon backbone. The nitrogen containing homologous chain corresponds to *HCN* polymers that can form from diaminomaleonitrile (DAMN) tetramers in aqueous solutions in the presence of an unsaturated complex matrix (Ruiz-Bermejo et al. 2012). The oxygen-based polymer was an amphiphilic polyethylene glycol (PEG). Since PEG is a polyether, it is not clear how this polymer can form in an aqueous solution.

## Methods

### Experimental procedure

**Fig. 1:**
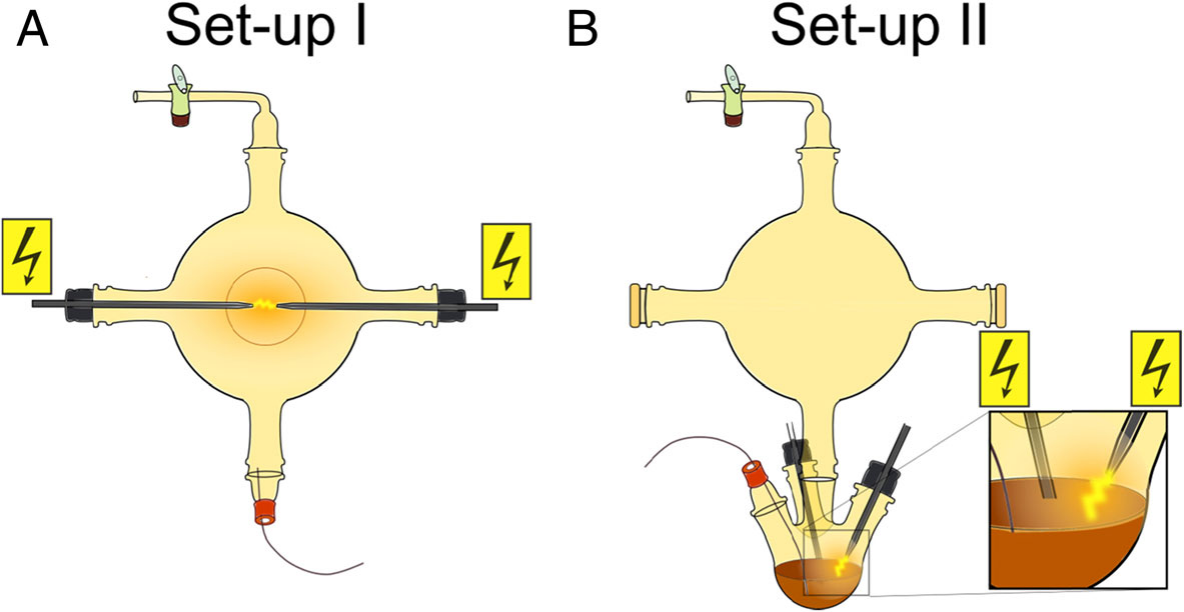
Experimental set-ups. a) Electric discharge in the gaseous phase; b) electric discharge onto the water surface

We used two different set-ups (Fig. 1) to perform Miller-Urey-type experiments. Set-up *I* consisted of only one *5 l*- or *1 l*-flask while set-up *II* was made of a *5 l*-flask on top of a *1 l*-vessel. The main difference between the two set-ups was the positioning of the electrodes. In set-up *I* we had an electric discharge in the gaseous phase, whereas in set-up *II* we sparked directly onto the water-surface. For sparking (~*10 kV* – *12 kV* sawtooth, *20 Hz*, ~ *20 W*, direct current), we used a home-made high-voltage device (based upon a transformer from a cathode ray tube, max. *25 kV*). For experiments, either set-up was filled with *200 ml* ultrapure water (Sartorius stedim biotech, arium *611UV* or GENO, Grünbeck) heated to *85* °*C* - *95* °*C*. The initial gas phase consisted of methane (purity *2.5*, Praxair), ammonia (purity *3.8*, Praxair), and water vapor in a ratio of *7*:*2*:*1*. The pressure in the closed system was about *1 atm*, but varied slightly due to the reactions. Usually, the experiment ran for *2* to *4* days. Samples were extracted and measured either at fixed points in time or in real-time. For storage, we lyophilized the samples immediately after extraction.

We performed several runs to check for reproducibility (Table 1). We tried to keep the time between extraction and analysis as short as possible. *A1*, *A2*, *A3* and *A4* were produced in set-up *I*, *B1* and *B2* in set-up *II*. In experiments *A2*, *A3* and *A4*, we extracted several samples at consecutive time points and numbered them with increasing integers, as detailed in Table 1. In *A3*, we withdrew both gaseous and liquid samples simultaneously, which we labeled with the subscripts “gas” and “liquid”, respectively. To check the influence of different substances, we added ethanolamine (*HOC*_*2*_ *H*_*4*_ *NH*_*2*_) in the course of *B2*, hydrogen peroxide (*H*_*2*_ *O*_*2*_) before the extraction of *A3*_*1*, phosphoric acid between *A3*_*1* and *A3*_*2* and hydrogen peroxide between the extractions of *A4*_*2* and *A4*_*3*. We observed the formation of polyethylene glycol (PEG) during the experiments. Samples *B1*, *B2*, *A2*_*2*, *A3*_*1*_*liquid*_, *A3*_*2*_*liquid*_, *A4*_*1* and *A4*_*3* contained PEG oligomers (marked by (+)). Samples where we could not detect PEG were marked by (−).

**Table 1:** Overview of sample preparation details and techniques used for analysis. Each row symbolizes a different experiment

Sample	Set-up type used	Time point of extraction	State of matter	Obser-vation of PEG	Analytical methods used	remarks
*A1*-	*I*	*51.5 h*	liquid, lyophilized	−	NMR, CARS	
*A2*_*1*-	*I*	*20.9 h*	liquid, lyophilized	−	NMR	Use of ^*13*^ *C*-methane and ^*15*^ *N*-ammonia.
*A2*_*2*+	*I*	*27.1 h*	liquid, lyophilized	+	NMR	Use of ^*13*^ *C*-methane and ^*15*^ *N*-ammonia.
*A3*_*1*_*liquid*_+	*I*	*2.5 h*	liquid	+	GC/MS	Injection of *50 nmol* hydrogen peroxide (*H*_*2*_ *O*_*2*_) at *2.0 h*; extracted with diethylether : n-pentane : cyclohexane = *1* : *1* : *1*.
*A3*_*1*_*gas*_-	*I*	*2.5 h*	gaseous	−	GC/MS	Injection of *50 nmol* hydrogen peroxide (*H*_*2*_ *O*_*2*_) at *2.0 h*.
*A3*_*2*_*liquid*_+	*I*	*27.5 h*	liquid	+	GC/MS	Injection of *50 nmol* phosphoric acid at *3.2 h*; extracted with diethylether : n-pentane : cyclohexane = *1* : *1* : *1*.
*A3*_*3*_*gas*_-	*I*	*27.5 h*	gaseous	−	GC/MS	Injection of *50 nmol* phosphoric acid at *3.2 h*.
*A3*_*3*_*liquid*_-	*I*	*95.8 h*	liquid	−	GC/MS	Stop of electric discharge at *46.8 h*; extracted with diethylether : n-pentane : cyclohexane = *1* : *1* : *1*.
*A3*_*3*_*gas*_-	*I*	*95.8 h*	gaseous	−	GC/MS	Stop of electric discharge at *46.8 h*.
*A4*_*1*+	*I*	*2.5 h*	liquid, lyophilized	+	GCxGC/MS	Extracted with n-hexane.
*A4*_*2*+	*I*	*23.0 h*	liquid, lyophilized	−	GCxGC/MS	Extracted with n-hexane.
*A4*_*3*+	*I*	*46.0 h*	liquid, lyophilized	+	GCxGC/MS	Injection of *50 nmol* hydrogen peroxide (*H*_*2*_ *O*_*2*_) at *44.3 h* and *45.6 h*; extracted with n-hexane.
*B1*+	*II*	*52.0 h*	liquid, lyophilized	+	NMR	
*B2*+	*II*	*53.8 h*	liquid, lyophilized	+	NMR, CARS	Injection of *50 nmol* ethanolamine (*HOC*_*2*_ *H*_*4*_ *NH*_*2*_) at *5.2 h*, *23.2 h*, *30.2 h* and *45.6 h*.

### Analytical techniques

#### NMR spectroscopy

Sample volumes of *40 ml* from samples *A1*, *A2*, *B1*, and *B2* were lyophilized immediately after extraction, each resulting in a dry weight of about *26 mg* that equals a concentration of *0.65 g*/*L* (Table 1). The samples were redissolved in deuterated water, and undissolved sediments were separated by centrifugation. The supernatant was analyzed at *290 K* on either a *600 MHz* or *800 MHz* spectrometer (Bruker Avance III), recording *1*D-diffusion ordered spectroscopy (DOSY) (implementing stimulated-echo and LED correction), *2*D-rotating frame nuclear Overhauser effect spectroscopy (ROESY) (mixing time 450 ms), distortionless enhancement by polarization transfer (DEPT) edited ^*13*^ *C*, ^*1*^ *H*-heteronuclear single quantum coherence (HSQC), and sensitivity enhanced heteronuclear multiple-bond correlation (HMBC) spectra.

ROESY experiments were recorded using *256* increments and *96* scans per experiment. ^*13*^ *C*-HSQC and HMBC spectra were recorded with *128* increments, encoding a sweep-width of *200 ppm* in the ^*13*^ *C* dimension.

DOSY experiments were calibrated using a mixture of molecules from *250* to *2000 Da*. The optimized values for *Δ* and *δ* delays were *50 ms* and *4.6 ms*, respectively at *800.13 MHz*, and *50 ms* and *4.4 ms* at *600.13 MHz*. DOSY experiments were recorded with *128* or *256* scans for each point, with a variation of gradient strength from *2* to *95*%.

#### CARS spectroscopy

Sample volumes of *40 ml* from samples *A1*- and *B2*+ were lyophilized immediately after extraction, each resulting in a dry weight of about *26 mg* that equals a concentration of *0.65 g*/*L*. Each sample was resuspended in *1 ml* deionized water. Solid particles in the suspensions were size-separated and removed with a *0.22*-*μm* syringe filter. *10 μl* of the resulting sample solution were placed between two cover slips, separated by a *100*-*μm* spacer.

Experiments were carried out using a home-built, multiplex-CARS microspectrometer (Gomes da Costa 2010): A mode-locked Ti:sapphire laser oscillator (Mira *900*-P, Coherent Inc.), providing a pulse train of *2.3*-*ps* pulses at a repetition rate of *76 MHz* and a wavelength of *808 nm*, was split into two parts. One part served as the pump pulses, while the other part was coupled into a photonic crystal fibre (PCF: femtowhite*800*, NKT photonics) generating a picosecond supercontinuum. The long-wavelength part of the latter was used as Stokes-pulses for CARS. The pump and Stokes beams were separately controlled in size and collimation by telescopes, and recombined on a dichroic mirror (zq *800* rdc, Chroma Technology). The spatially and temporally overlapped pump and Stokes pulses were collinearly focused into the sample using a *1.2*-N.A. water immersion objective (UPLSAPO IR *60* W, Olympus). The average pump and Stokes powers in the focus amounted to respectively *60 mW* and *13 mW*. The generated CARS emission was par-focally collected in the forward-direction with an identical objective and spectrally isolated from the excitation pulses with two short-pass filters (RU*785*SP, Semrock and FES*800*, Thorlabs). Spectrally resolved detection was performed using an imaging spectrometer, consisting of a *150*-*mm* monochromator (SP*150*, Acton Research) and a liquid-*N*_*2*_ cooled CCD-array detector (Spec*10*, Roper Scientific). CARS spectra were recorded with total acquisition times of *50 s*.

All raw spectra were first subjected to cosmic ray removal, detector dark-count subtraction, and corrected for broadband, intra-Stokes CARS contributions, independently recorded when the pump and Stokes pulses did not temporally overlap. Subsequently, CARS spectra taken inside the prebiotic broth sample solutions and in a reference sample of pure water were normalized with nonresonant CARS spectra recorded under identical experimental conditions when focused into the glass cover slip. For each normalized CARS spectrum, the vibrational phase spectrum was then retrieved by applying the maximum-entropy method (MEM), which included polynomial error-phase estimation within a subset of vibrationally nonresonant spectral regions (Vartiainen 1992). By using the recovered MEM-phase spectrum, its error-phase estimation, and the corresponding normalized CARS spectrum, the imaginary part of the vibrationally resonant, third-order susceptibility spectrum was reconstructed, resulting in the full characterization of the sample’s spectral Raman response, i.e., *Im*[*χ* ^(*3*)^(*ν*)] . Finally, the measured and reconstructed *Im*[*χ* ^(*3*)^(*ν*)] spectra of pure water were subtracted from that of the prebiotic broth sample solutions according to its weighted amplitude contribution at *3426 cm* ^−*1*^.

For an estimation of the number of vibrational modes inside our sample volume of *0.1* femtoliter, which is given by the diffraction limited focus dimensions, we employed the *Im*[*χ* ^(*3*)^(*ν*)] amplitude of the *CH*_*3*_-stretching peak intensity at *2940 cm* ^−*1*^. Using DMSO in aqueous solution as a reference, the observed peak intensities of the prebiotic broth samples typically correspond to a range of *0.7 × 10* ^*6*^ to *20 × 10* ^*6*^ of *CH*-stretching vibrational modes inside the probe volume. If the signal was due to DMSO with two *CH*_*3*_-stretching modes per molecule, this would indicate a molar concentration range of *10* to *300 mM* in the sample probed. For the complex mixture of diverse molecules with unknown number of *CH*-stretching bonds in the prebiotic broth samples, no absolute molar concentrations can be provided.

#### GC/MS

GC/MS consisted of a Clarus *500* GC (Perkin Elmer), equipped with a Zebron ZB-FFAP column (Phenomenex), and a quadrupole Clarus *500* MS (Perkin Elmer). The *15*-*m* long column had an inner diameter of *320 μm*. The elapsed time during measurement was *32* min. We applied a temperature gradient from *40* to *230*°, increasing in a stepwise manner by *6*° per minute. The spectra of the detected molecules were compared to the NIST database.

The gaseous samples (*A3*_*1*_*gas*_-, *A3*_*2*_*gas*_-, and *A3*_*3*_*gas*_-) were directly injected into the gas chromatograph. Hydrophobic substances were extracted from *1 ml* liquid sample that included the oil-like layer in *1 ml* of an *1*:*1*:*1*-mixture of dethylether, *n*-pentane and cyclohexane (*A3*_*1*_*liquid*_+, *A3*_*2*_*liquid*_+, and *A3*_*3*_*liquid*_-).

By comparing the surface area of the peaks in the chromatogram, we determined the concentrations of substances in the liquid phase relative to a reference (Appendix: Tab. 6, 7, and 8). We found the solvent cyclohexane most suitable to use as a reference. The estimated concentrations of the different molecules detected in the broth reached up to the millimolar range.

#### GCxGC/MS

The samples *A4*_*1*, *A4*_*2*, and *A4*_*3* were analyzed with a comprehensive two-dimensional gas chromatograph coupled with a quadrupole mass spectrometer (GCxGC-EI-qMS). The lyophilized samples were extracted with water and n-hexane (*1*:*1*). Afterwards the organic phase was diluted by a factor of *5* with *n*-hexane. A procedural blank was also prepared. The samples were introduced into the GCxGC at an injection temperature of *310* °*C* and a split-ratio of *1*:*5*. The temperature program started from *60* °*C* (*1 min* hold time) with a gradient of *5* °*C*/*min* up to *200* °*C*, then with *3* °*C*/*min* up to *300* °*C* (*1 min* hold time). For the first dimension, a non-polar Phenomenex ZB-5MSi (*30 m × 0.25 mm × 0.25 μm*) column was used. The second dimension separation was performed on a middle-polar Phenomenex ZB-50 (*2.5 m × 0.1 mm × 0.1 μm*$) column. Helium was used as a carrier gas with a velocity of *1.96 ml*/*min*. The modulation time was set to *3.8 s*. The interface temperature of the transfer line was *310* °*C*. For the electron impact ionization the ion source temperature was set to 200 °C, the electron energy was *70 eV* and a scan range from *40 to 800 m*/*z* was used. The data were evaluated with the NIST/W9N08 databases.

By standard addition we determined the production of (*1.26* ± *0.08*)*μg C*_*17*_-linear alkane and (*1.66* ± *0.17*) *μg* linear *C*_*19*_-alkane in *5 ml* reaction product. That equals concentrations of (*1.048* ± *0.067*)*μM* and (1.236 ± 0.127)*μ*M, respectively.

## Results

### NMR spectroscopy

**Fig. 2:**
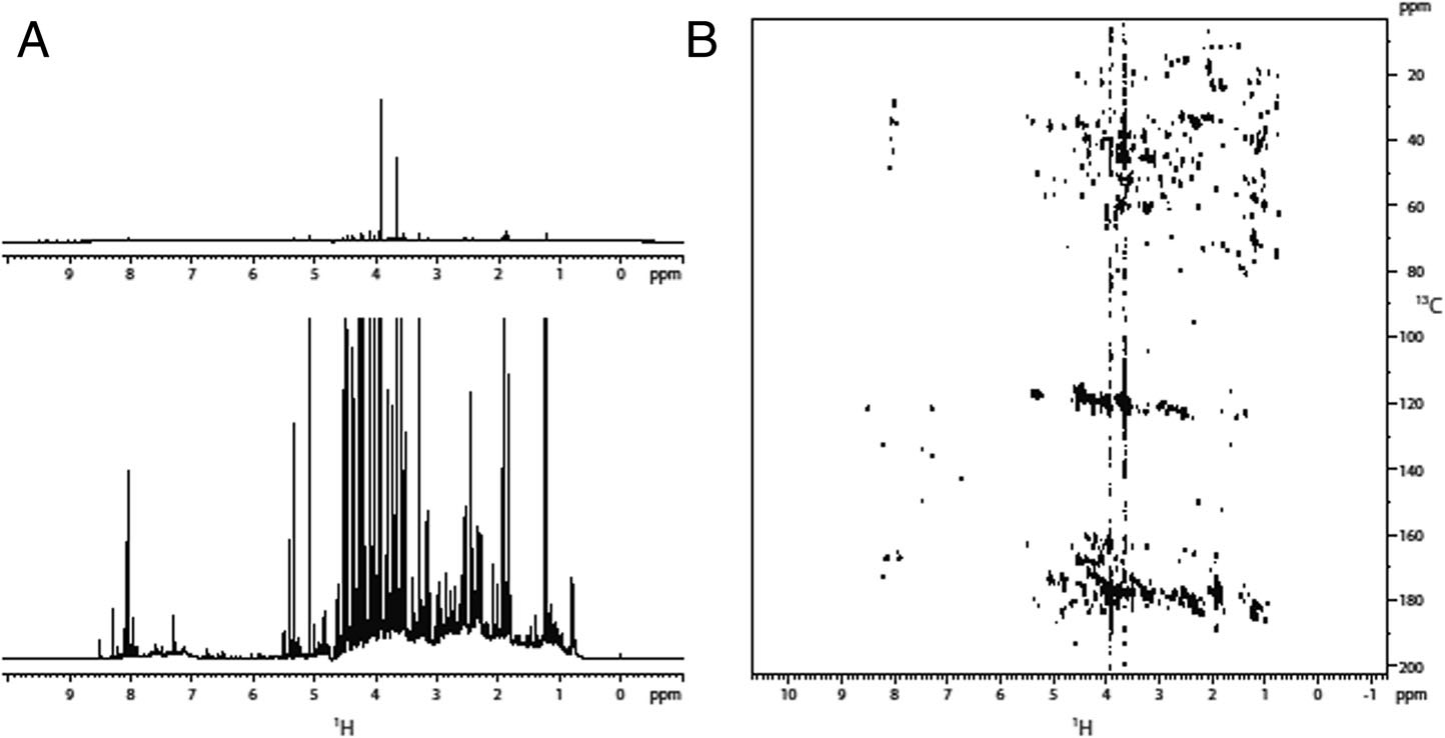
Sample *A1*-. A) Full view of the ^*1*^ *H* spectrum (top) and detailed view of the minor species observed (bottom); B) HMBC spectrum

*A1*- revealed a broad distribution of ^*1*^ *H* resonances between *9.6 ppm* and *0.5 ppm* (Fig. 2). The most intense signals were two singlets at *4.0* and *3.7 ppm*. DOSY experiments showed a uniform distribution of diffusion coefficients between −*9* and −*9.5 m* ^*2*^/*s*, which corresponds to molecules of molecular weight (MW) in the range from *250* to *500 Da*. DEPT edited HSQC showed carbons in a span from *10* to *80 ppm* corresponding to *CH*, *CH*_*2*_ and *CH*_*3*_ aliphatic groups (alkanes, alkenes and alkynes with various substitutions including heteroatoms). Additional carbon resonances in the range from *110* to *140 ppm*, correlating with ^*1*^ *H*s between *8.3* and *5.8* suggested the presence of differently substituted alkene and aromatic moieties. A signal at ^*13*^ *C* (*166.8 ppm*) and ^*1*^ *H* (*8.0 ppm*) suggested the presence of formamide groups; the group correlated with a carbon at *28.9 ppm* in the HMBC spectrum, which was compatible with a *N*-methyl-formamide (Fig. 6). The HMBC spectrum showed correlation between aliphatic carbons and carbons between *115* and *125 ppm* (alkenes, cyanides, cyanamides) and between *150* and *190 ppm* (esters, amides, acids and heteroatom substituted alkenes).

**Fig. 3:**
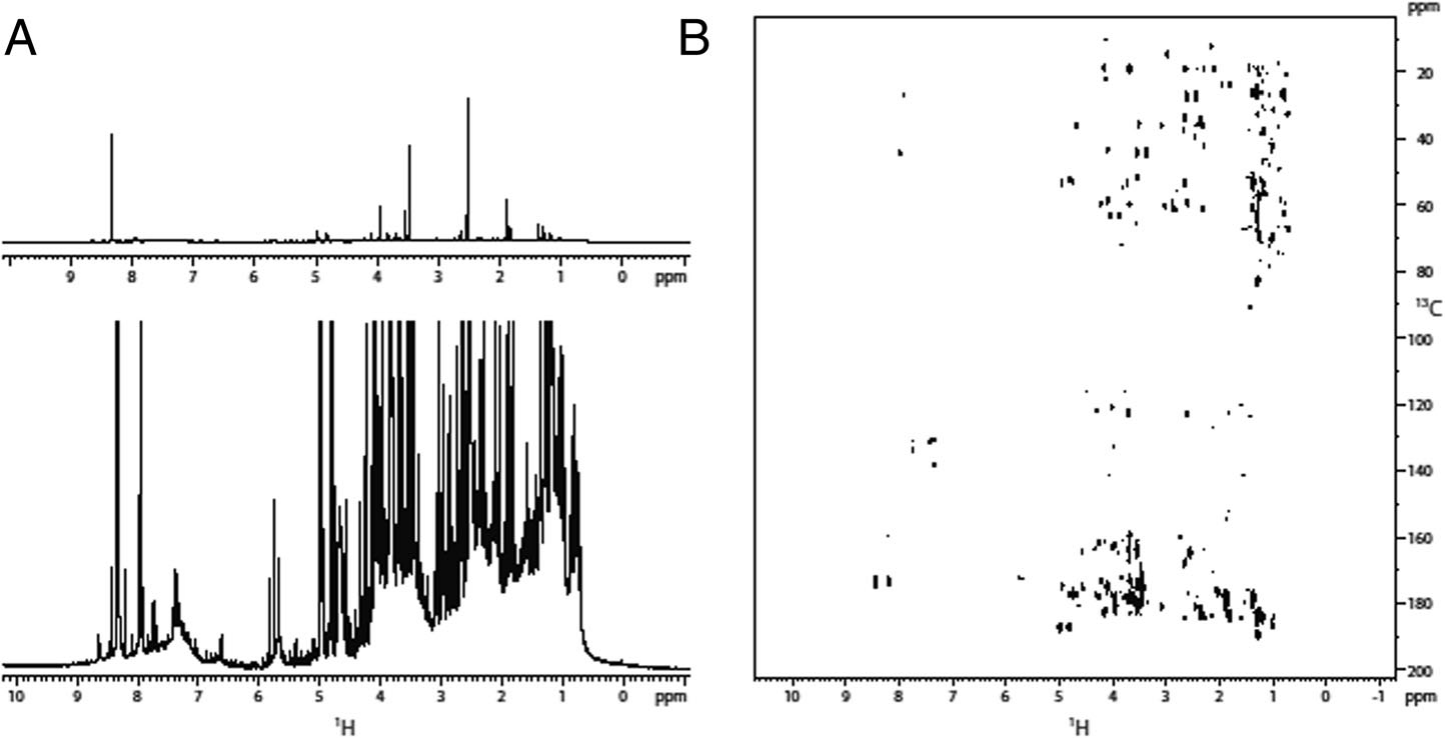
Sample *B1*+. A) Full view of the ^*1*^ *H* spectrum (top) and detailed view of the minor species observed (bottom); B) HMBC spectrum

Similarly to sample *A1*-, the ^*1*^ *H 1*D spectrum of sample *B1*+ showed resonances with chemical shifts in the range from *9.0* to *0.5 ppm* (Fig. 3). The most intense resonances were singlets at *8.41*, *3.55*, and *2.6 ppm*.

DEPT edited HSQC and HMBC spectra were qualitatively similar to those of *A1*-. The intense signal at *2.6 ppm* correlates to a carbon at *27.4 ppm*, compatible with a methylamine (Fig. 6). The singlet at *3.55 ppm* was bound to a carbon at *44.1 ppm* and showed an HMBC connection to a carbon at *175 ppm*. The network of resonances was compatible with a glycine (Fig. 6). Similarly the methylene group at *4.0 ppm*, connecting with a carbon at *63.3 ppm* in the HSQC and with a carbon at *180.6 ppm* in the HMBC, may correspond to a *2*-hydroxyacetic acid (Fig. 6). Finally the resonance at *8.4 ppm* directly connected with a ^13^C at *173.8 ppm* can be attributed to formamide (Fig. 6).

**Fig. 4:**
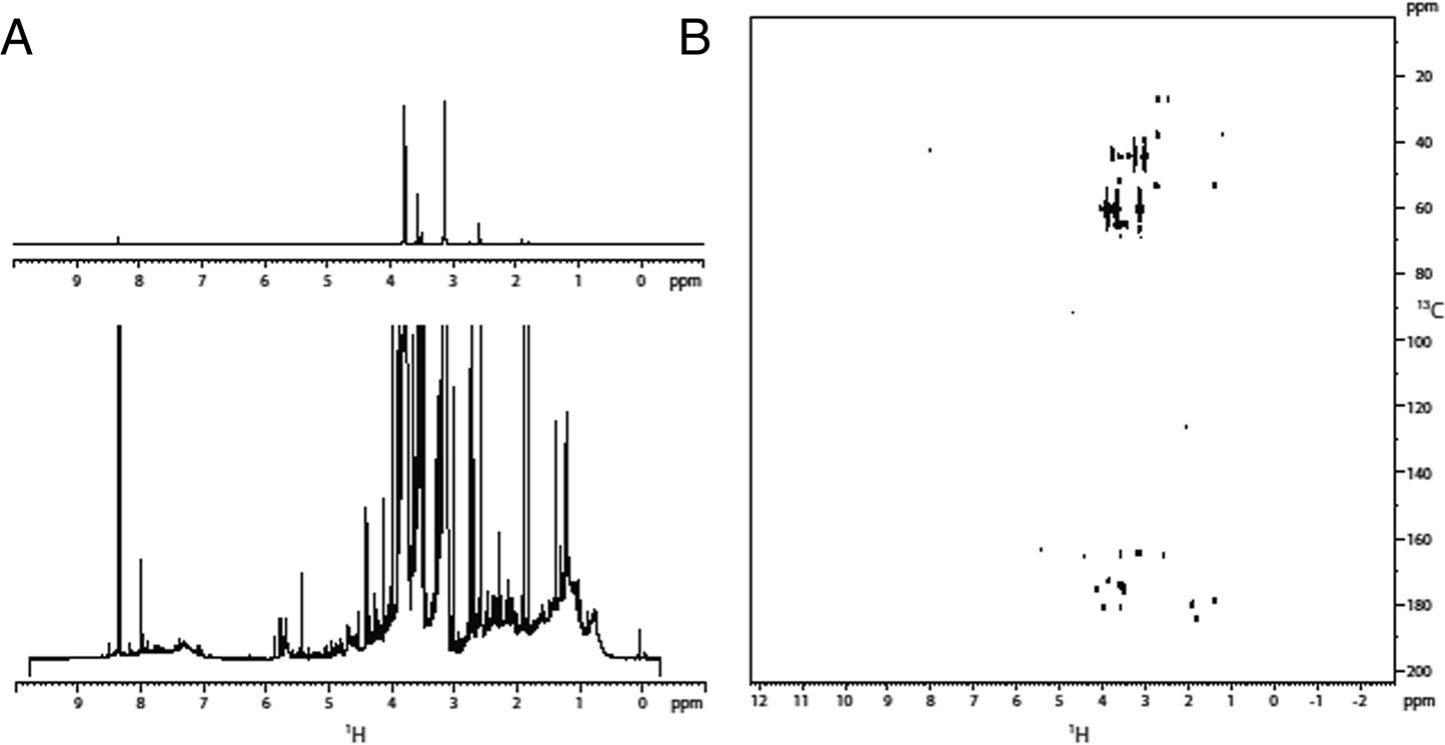
Sample *B2*+. A) Full view of the ^*1*^ *H* spectrum (top) and detailed view of the minor species observed (bottom); B) HMBC spectrum

The ^1^H *1*D spectrum of sample *B2*+ shows two intense resonances at *3.88* and *3.24 ppm* (doublets of doublets) within a distribution of resonances between *0.0* and *9.4 ppm* (Fig. 4). As in the samples *A1*- and *B1*+, the DOSY experiment did not reveal the presence of polymers. The DEPT edited HSQC showed two very intense correlations at *44.2* (*δ* ^*1*^ *H 3.24*) and *60.42* (δ ^1^H 3.88) *ppm* together with a number of much less intense correlations including the one assigned to the formamide derivative in sample *B1*+. The proton resonance ^*1*^ *H* at *3.24 ppm* correlated in the HMBC with a carbon at *164.4 ppm*, allowing to hypothesize the presence of a (*2*-hydroxyethyl)urea molecule (Fig. 6).

**Fig. 5:**
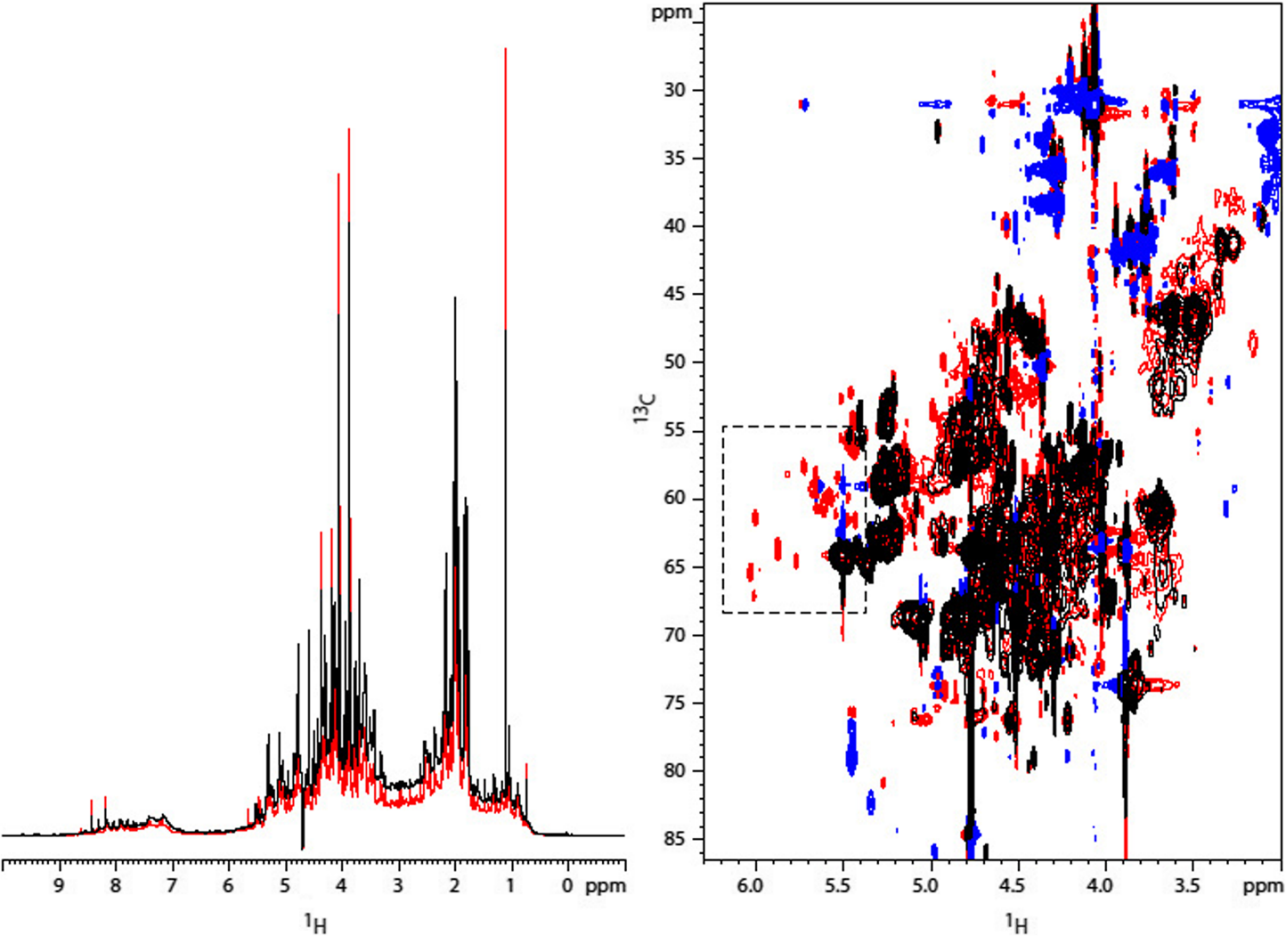
Left: comparison of the ^*1*^ *H* spectra for samples *A2*_*1*- (black) and *A2*_*2*+ (red). Right: a detail of the comparison between ^*13*^ *C*-edited HSQC spectra. Methylene groups are depicted in black for sample *A2*_*1*- and in red for sample *A2*_*2*+. Methyl groups are colored in blue for both samples. The dashed square marks unique methylene groups observed in sample *A2*_*2*+

The ^*1*^ *H 1*D spectrum of sample *A2*_*1*- showed resonances between *0* and *9 ppm*, as all the other samples (Fig. 5). The DEPT edited HSQC showed a majority of *CH*_*2*_ groups between *45* and *80 ppm* correlating with protons between *3.5* and *5.5 ppm*. These corresponds to heteroatom-substituted alkanes and alkenes, and conjugated alkenes. In the aromatic region corresponding to ^*1*^ *H* from *6.5* to *8.5 ppm* and ^*13*^ *C* from *120* to *140 ppm*, we observed only two signals. Additional *CH* groups were seen at ^*13*^ *C* from *162* to *176 ppm* and ^*1*^ *H* from *7.8* to *8.3 ppm*. These correspond among others to substituted formic acid, formamide, and methanimine.

**Fig. 6:**
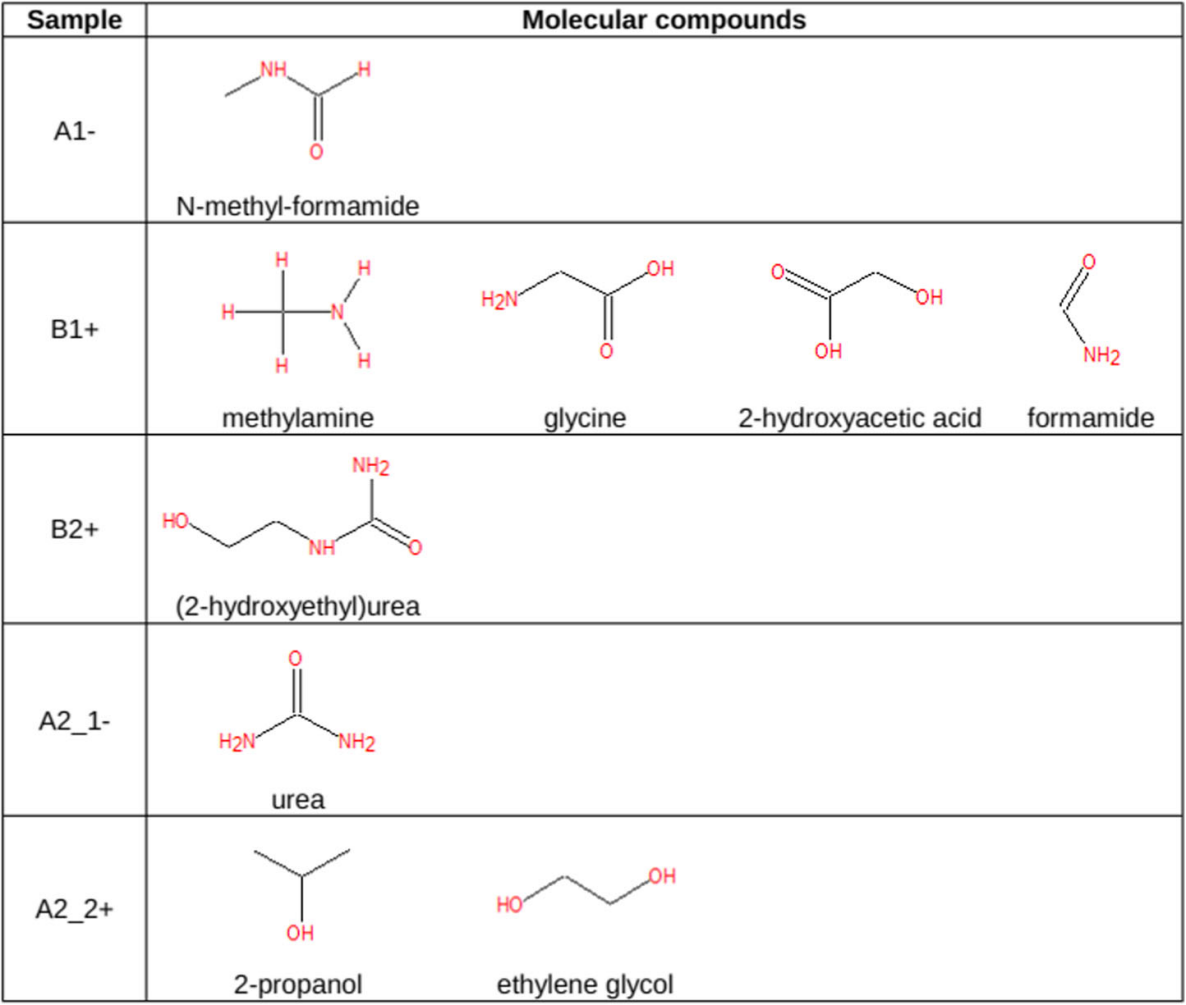
Molecules detected in samples *A1*-, *B1*+, *B2*+, *A2*_*1*-, and *A2*_*2*+

The ^*15*^ *N*, ^*1*^ *H*-HSQC of sample *A2*_*1*- showed several peaks in the range from *130* to *100 ppm* (^*15*^ *N*) and from *9* to *6.5 ppm* (^*1*^ *H*). This was compatible with the presence of amines, amides, and terminal azides (Loewenstein 1982; Forman 1963). A single resonance at ^*15*^ *N 73.7 ppm* and ^*1*^ *H 6.7 ppm* could be ascribed to urea (Fig. 6).

The HMBC ^*13*^ *C* spectrum showed that the majority of the *CH*_*3*_ groups correlated with quaternary carbons between *170* and *190 ppm* (esters, amides, aldehydes). A second group of connections was between *CH*_*2*_ moieties ranging from *3.8* to *4.5 ppm* and ^*13*^ *C* atoms at around *120 ppm*. Here, we expected alkenes, isocyanate, and nitrile moieties.

Sample *A2*_*2*+ was similar to sample *A2*_*1*-. The ^*1*^ *H* resonances at *4.3*, *4.0*, *3.9* and *1.1 ppm* were of higher relative intensity in *A2*_*2*+ as compared to *A2*_*1*-. The ^*1*^ *H* resonances observed at *4.3*, *4.0*, and *3.9 ppm* were either methyl or methine groups and correlated with ^*13*^ *C* carbons at *50.3*, *30.9*, and *63.2 ppm*, respectively. In the HMBC, the ^*1*^ *H* at *4.3 ppm* correlated with a carbon at *121.3 ppm*. The ^*1*^ *H* at *4.0 ppm* correlated with a carbon at *53.6 ppm* (*CH*_*2*_). The ^*1*^ *H* at *4.3 ppm* correlated with a carbon at *121.3*~*ppm*. The ^*1*^ *H* at *4.0 ppm* also correlated with carbons at *53.6* (*CH*_*2*_ with ^*1*^ *H* at *4.75 ppm*), *73.9* (*CH*_*2*_ with ^*1*^ *H* at *3.8 ppm*), *120.1*, *163.2*, and *186.9 ppm*. The ^*1*^ *H* at *3.9 ppm* correlated with a carbon at *180.8 ppm*. The ^*1*^ *H* resonance at *1.1 ppm* was bound to a carbon at *32 ppm*, and correlated in the HMBC to another carbon at *72.5 ppm*. The spin system was compatible with *2*-propanol (Fig. 6). However the fact that this molecule was not enriched in ^*13*^ *C* (no ^*1*^ *J*_*HC*_ coupling) in *1*D spectra indicated that the *2*-propanol did not stem from a chemical reaction of the gases. Possible sources of ^*12*^ *C* were the cleaning agent deconex (Borer Chemie AG), or the teflon used for sealing.

In the ^*13*^ *C* HSQC spectra of sample *A2*_*2*+, we saw new *CH*_*2*_ moieties at *5.6* and *59.9 ppm* (Fig. 5), compatible with ethylene glycol, the monomer of PEG (Fig. 6).

The comparison of the ^*1*^ *H 1*D and the HMBC spectra of the different samples (Figs. 2, 3, 4, and 5), clearly indicates that the composition of the primordial broth varied strongly between the different experiments. The variability occurred even between samples extracted from the same experiment at different time points (Fig. 5).

Nevertheless, we detected many carbon moieties such as *CH3*, *CH2*, and *CH* in the aliphatic range. We observed a variety of molecules containing nitrogen moieties, for example in form of amides or *C*≡*N* triple bonds. Furthermore, many *C*=*O* bonds were detectable, especially *C*-*OH* as for example in alcohol or *C*-*O* double bonds in aldehydes, or the combination of both (acid moiety).

A commonality among the samples was that the molecules detected by NMR were located in a mass range between *250* and *500 Da*. The primordial broth mainly consists of molecules that are small relative to biomolecules like proteins.

### CARS spectroscopy

**Fig. 7:**
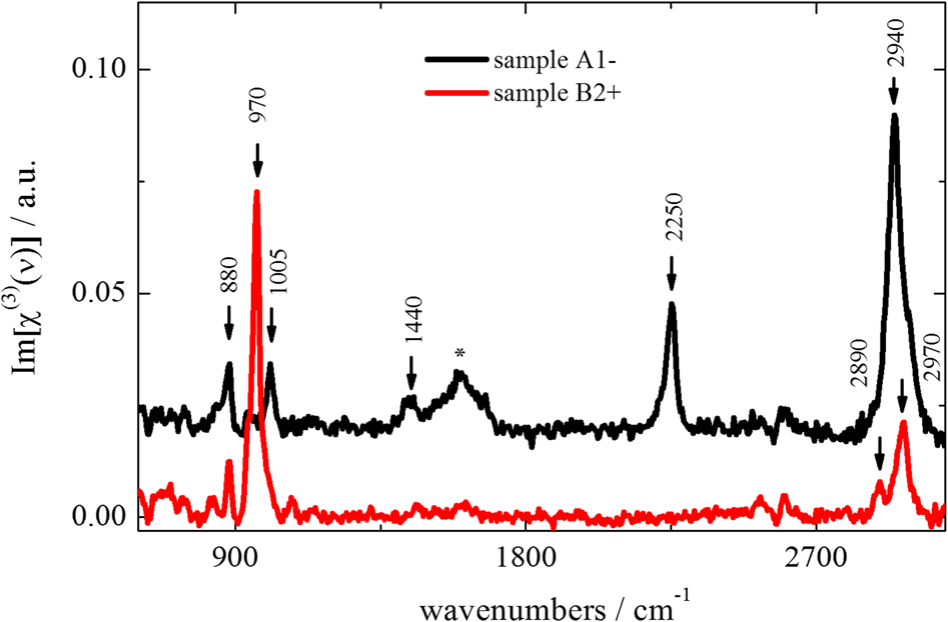
Reconstructed *Im*[*χ* ^(*3*)^(*ν*)] spectra of the primordial broth samples *A1*- (black curve) and *B2*+ (red curve) obtained from CARS spectroscopy experiments. The spectra are offset by *0.02*. See text for peak assignments

Figure 7 displays the *Im*[*χ* ^(*3*)^(*ν*)] spectra of samples *A1*- and *B2*+ as obtained by CARS measurements. Very distinct vibrational features were observed in the prebiotic broth sample *A1*- when compared to sample *B2*+.

The spectrum of sample *A1*- exhibited its most intense peak at *2940 cm* ^−*1*^ with shoulders appearing at *2890* and *2970 cm* ^−*1*^, assigned to the asymmetric *CH*_*2*_ stretching, symmetric *CH*_*3*_ stretching, and asymmetric *CH*_*3*_ stretching modes, respectively (Lin-Vien et al. 1991). While the bands in the *2800*–*3000 cm* ^−*1*^ region are ubiquitous to most organic compounds, the characteristic band observed at *2250* with a shoulder at *2210 cm* ^−*1*^ clearly indicated the presence of compounds that contained aliphatic (*R*_*aliphatic*_ − *C*≡*N*) and aromatic (*R*_*aromatic*_ − *C*≡*N*) triple bonds (Lin-Vien et al. 1991). In the so-called fingerprint region, the broad feature at about *1600 cm* ^−*1*^ (marked by ⁎) originated from the residual water band due to incomplete subtraction of the *Im*[*χ* ^(*3*)^(*ν*)] spectrum of pure water in that spectral range. The peaks at *1440*, *1005*, and *880 cm* ^−*1*^were assigned to the scissoring mode of the *CH*_*2*_ groups, the characteristic ring-breathing mode of phenyl residues, and the stretching mode of aliphatic *C*-*N* bonds, respectively (Lin-Vien et al. 1991). Alternatively, the *880*-*cm* ^−*1*^ band could also correspond to the *C*-*H* wag vibrations of *NH*_*2*_-monosubstituted benzene (Lin-Vien et al. 1991).

The most prominent differences observed in the spectrum of sample *B2*+ with respect to *A1*- were the lack of any detectable compounds that contain *C*≡*N* triple bonds and *CH*_*2*_ groups, and the existence of an additional narrow peak at *970 cm* ^−*1*^. The latter was tentatively assigned to breathing modes of small aromatic rings containing both *C* and *N* atoms (Bernard et al. 2006). This spectral feature pointed to a higher aromaticity of molecules in sample *B2*+ versus those in sample *A1*-, which was consistent with the lack of signatures for both aliphatic *CH*_*2*_ groups and aliphatic (*R*_*aliphatic*_ − *C*≡*N*) triple bonds. Similar vibrational spectral features had been observed for tholins (Imanaka et al. 2004; Bernard et al. 2006).

### GC/MS

The samples *A3*_*1*_*liquid*/*gas*_ to *A3*_*3*_*liquid*/*gas*_ were extracted at different time points during the same experimental run. The results of the GC/MS analysis are shown in tables 3–8 (see Appendix). In addition to the molecule name as well as molecular and structural formula, the hit ratio given by the NIST database, and the delay times of the GC are given. For suggested molecules with a low-percentage hit ratio, a second or even a third match is shown. We saw that the quantity of different detected molecules in the gaseous samples diminished (from *40* to *34* and *25* molecules) for samples *A3*_*1*_*gas*_-, *A3*_*2*_*gas*_-, and *A3*_*3*_*gas*_-, respectively, while the number increased (from *4* to *9* and *15* molecules) in the liquid samples for *A3*_*1*_*liquid*_+, *A3*_*2*_*liquid*_+, and A3_3_liquid_-, respectively. The ten most common molecular compounds of all gaseous and liquid samples are listed and compared in Table 2. For each sample the first number gives the quantity of the molecular compound being part of the first molecule matches in the NIST library. The numbers in brackets include second and third matches (Table 2). By far, the most frequent compounds were benzene rings, esters, and alkynes. In the gaseous samples, the quantity of a particular compound was either almost constant (alkynes, alkanes, ethers) for samples *A3*_*1*_*gas*_- to *A3*_*3*_*gas*_-, or descending (benzene rings, esters, carbonyls, alkenes, hydroxyls). Nitriles and amines constituted an exception. Their occurrence rose from *A3*_*1*_*gas*_- to *A3*_*2*_*gas*_- and fell from *A3*_*2*_*gas*_- to *A3*_*3*_*gas*_-. The quantity of one molecular compound in the liquid samples was conversely either constant (alkenes, ethers, hydroxyls) from *A3*_*1*_*liquid*_+ to *A3*_*3*_*liquid*_- or ascending (benzene rings, esters, alkanes, carbonyls). Esters were not detectable in *A3*_*1*_*liquid*_+ and *A3*_*2*_*liquid*_+, but they were found with 8 first matches in *A3*_*3*_*liquid*_-. Alkynes and amines were not detectable in the aqueous phase as first matches. As in the gaseous samples, nitriles were primarily found in the second probe (*A3*_*2*_*liquid*_+).

**Table 2:**
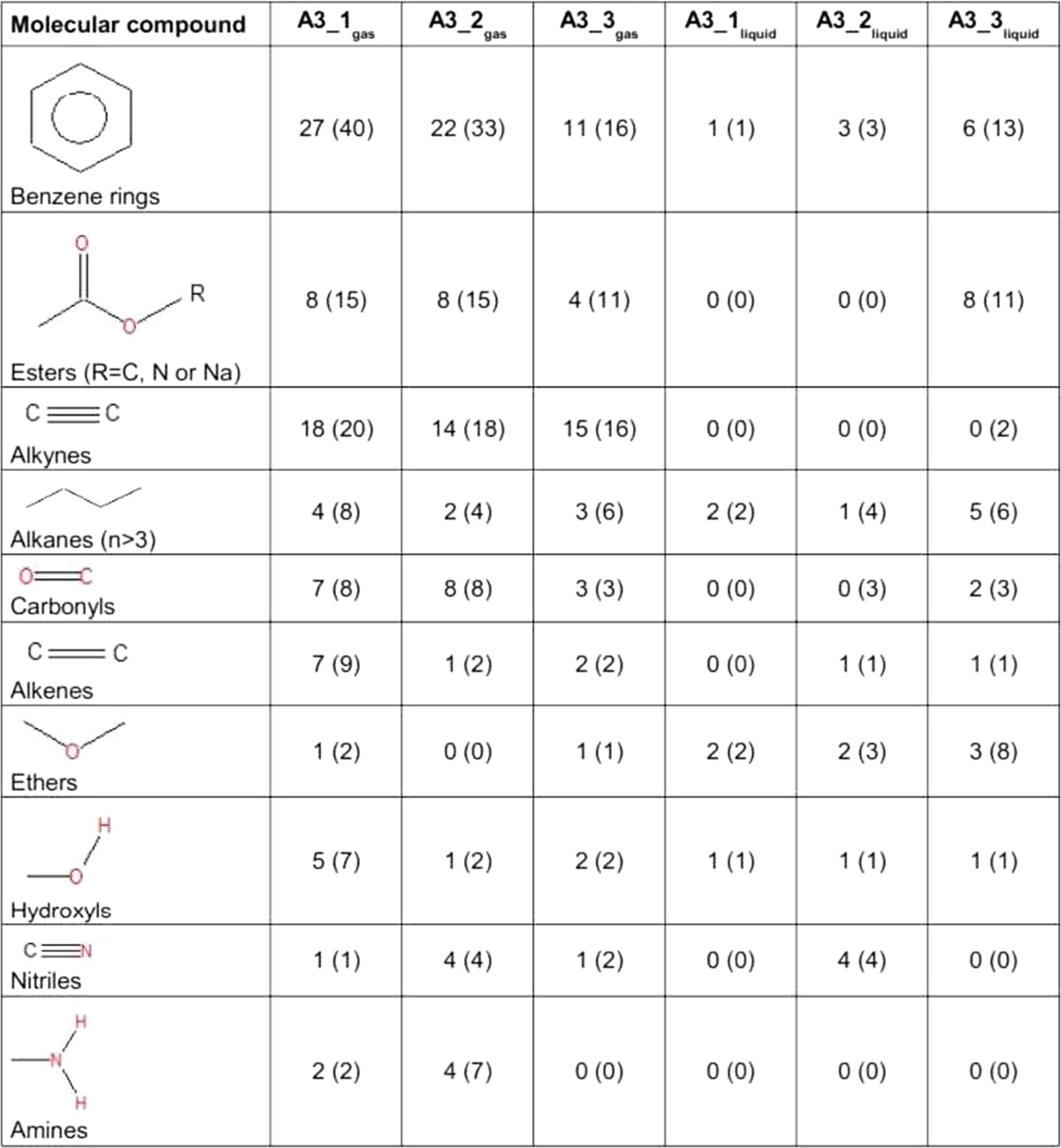
The first ten most common molecular compounds in samples *A3*_*1*_*liquid*/*gas*_ to *A3*_*3*_*liquid*/*gas*_ analyzed with GC/MS in descending order of abundance. The number indicates how many times the moiety occurs in the best matching molecule from the NIST database. The number in brackets indicates the number of occurences by including the second and third match

**Fig. 8:**
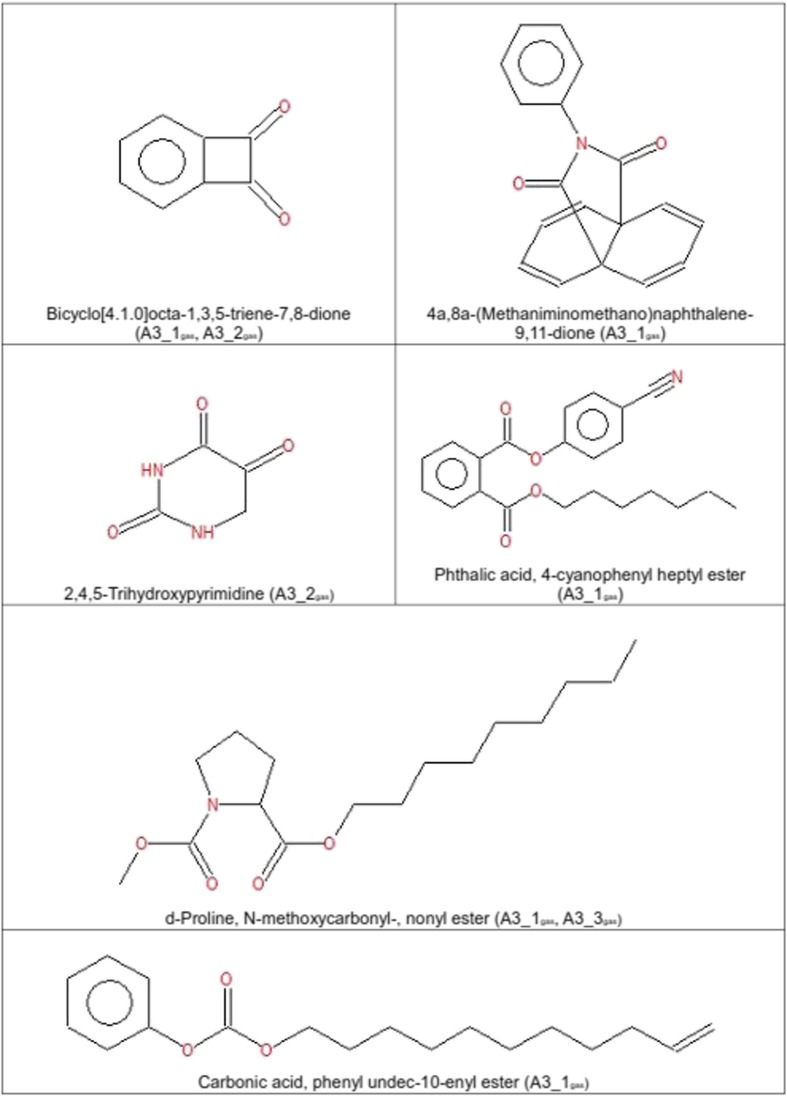
Molecules with distinct properties or highly reactive groups as detected in *A3*_*1*_*liquid*/*gas*_ to *A3*_*3*_*liquid*/*gas*_ by GC/MS

**Fig. 9:**
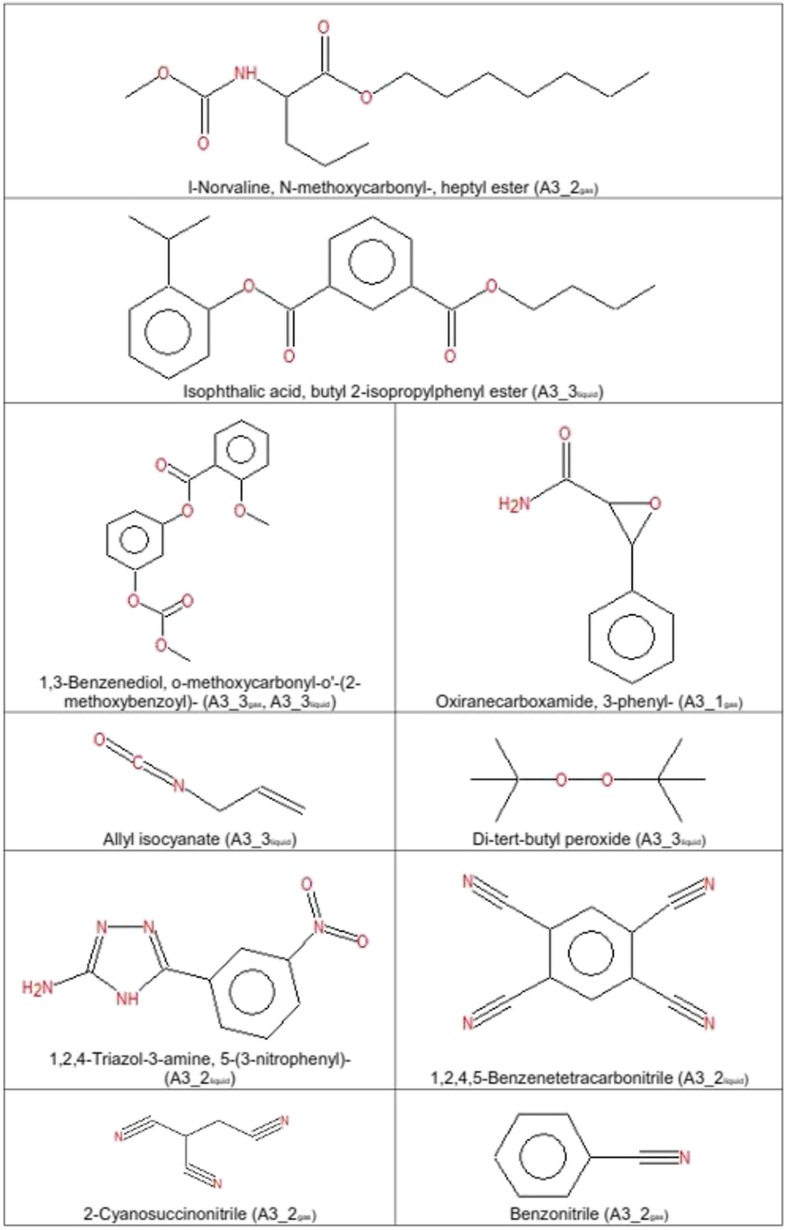
Molecules with special properties or highly reactive groups as detected in *A3*_*1*_*liquid*/*gas*_ to *A3*_*3*_*liquid*/*gas*_ by GC/MS

In Figs. 8 and 9, molecules with special properties or highly reactive functional groups are shown. In the gaseous samples, molecules with two ketone groups in a symmetrical order and compounds containing long carbon chains were detectable. In both phases, gaseous and liquid, we observed molecules with combinations of benzene rings and ester bonds as well as highly reactive functional groups like oxiranes, isocyanates, peroxides, nitro compounds, and nitrile groups.

### GCxGC/MS

**Fig 10:**
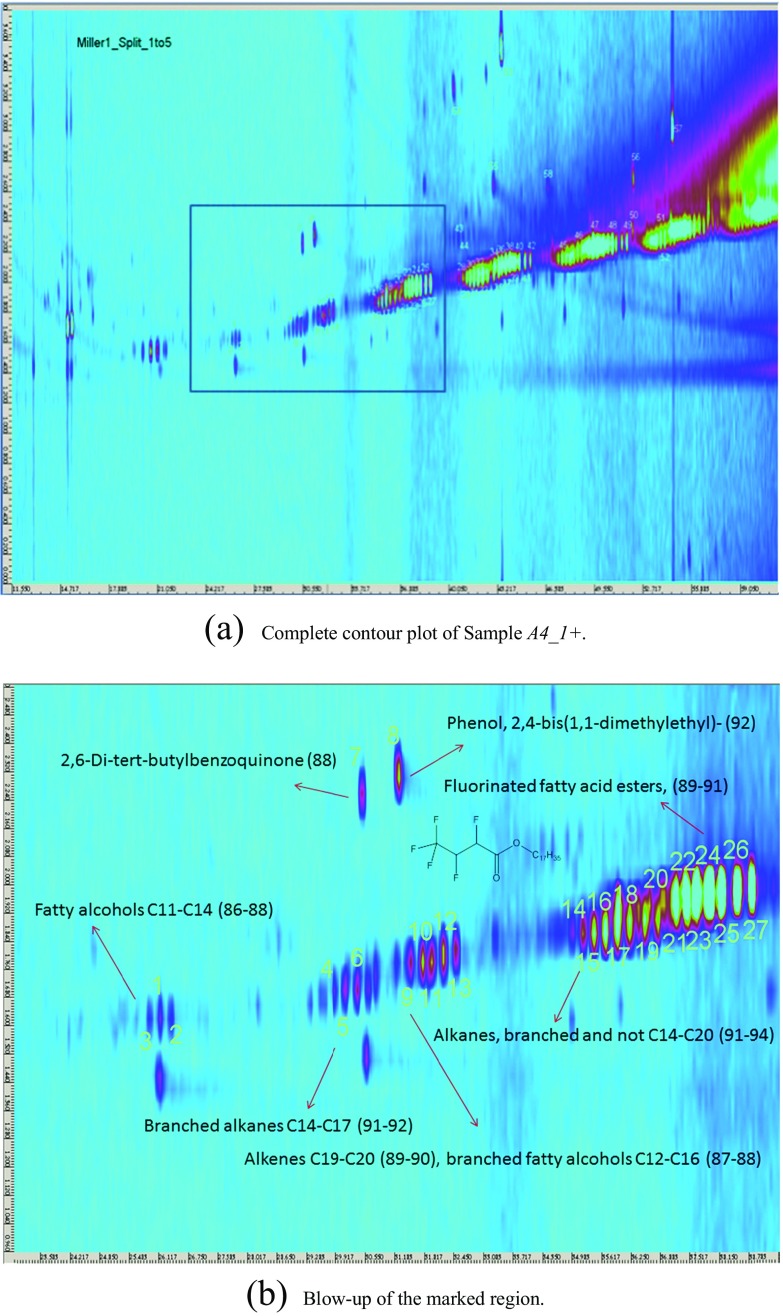
GCxGC/MS contour plot of Sample *A4*_*1*+ and blow-up of the marked region for a clearer demonstration of the spots

**Fig. 11:**
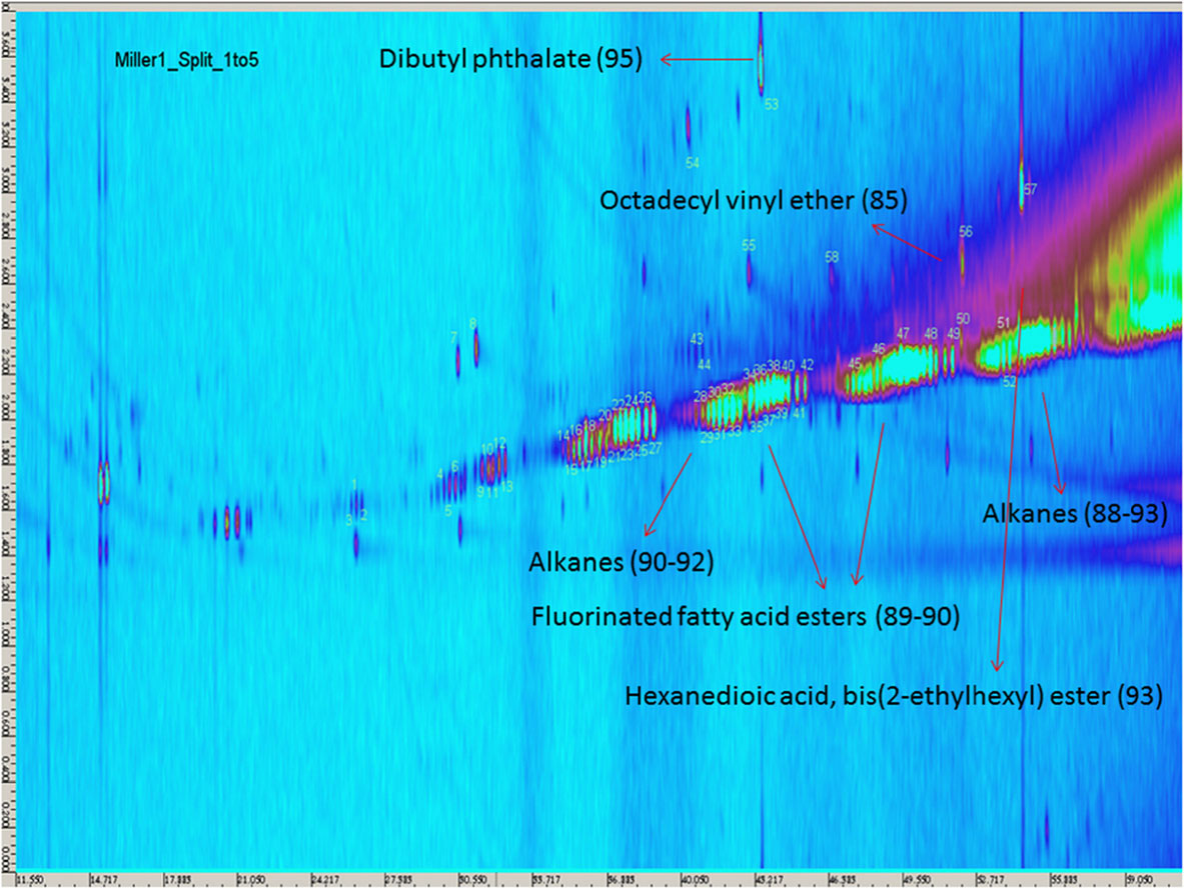
GCxGC/MS contour plot of Sample *A4*_*2*-

**Fig. 12:**
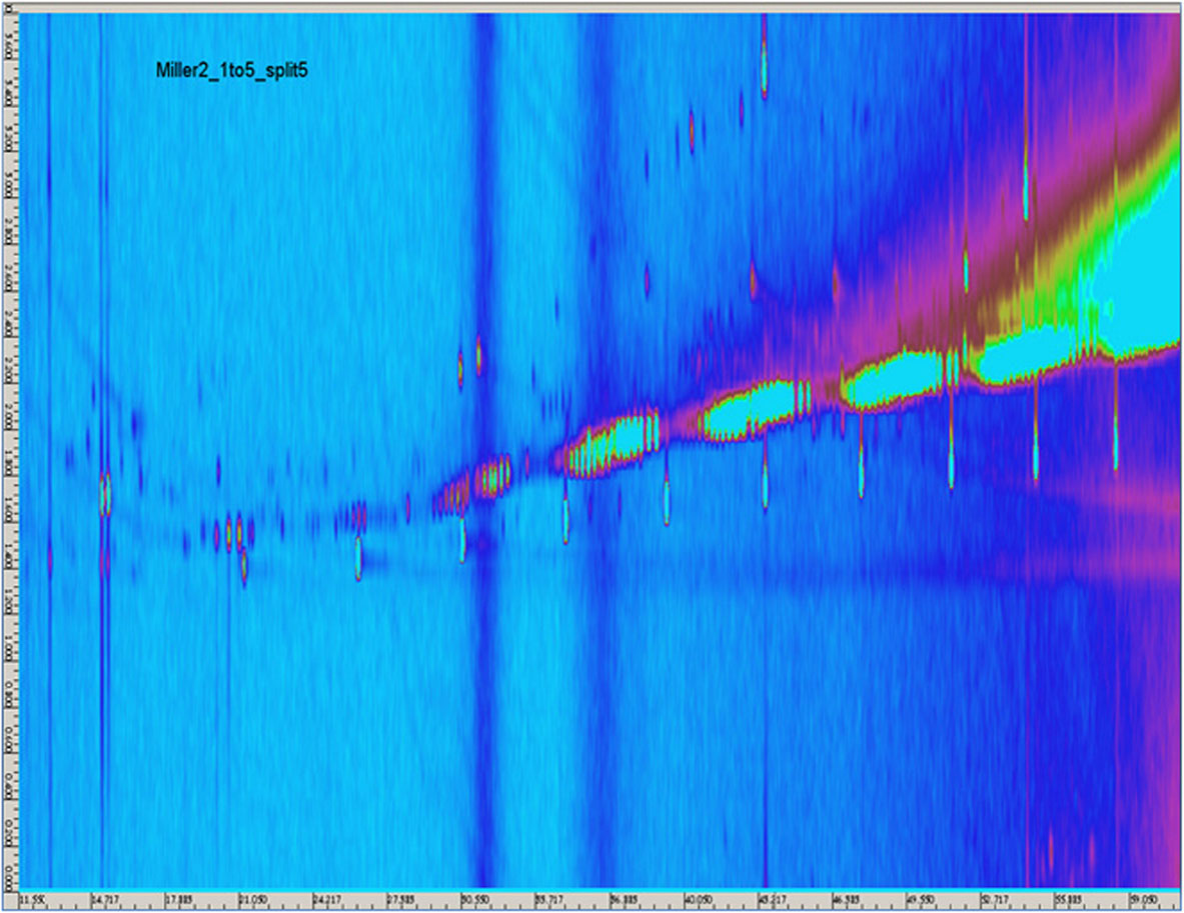
GCxGC/MS contour plot of Sample *A4*_*3*+

**Fig. 13:**
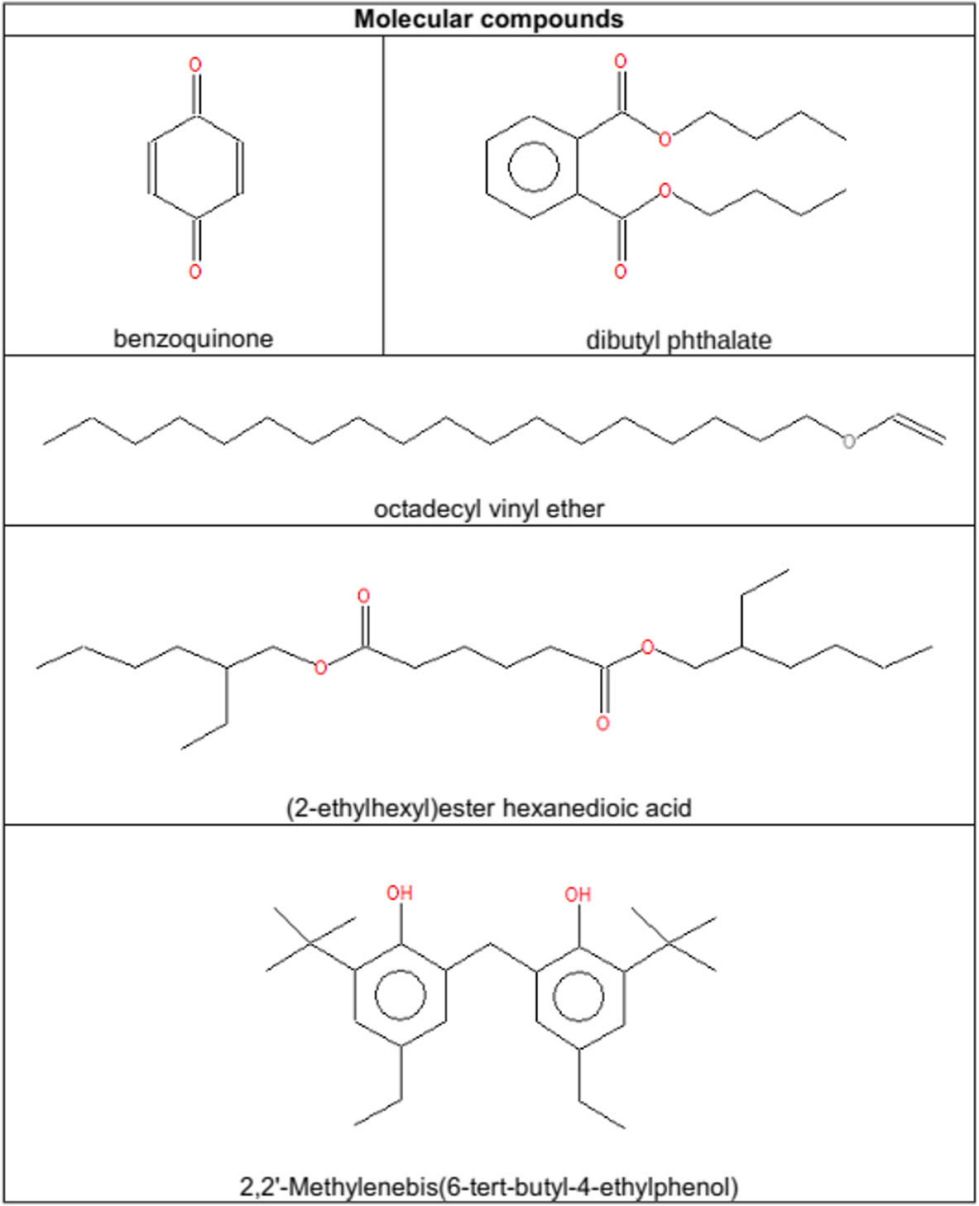
Molecular compounds detected by GCxGC/MS

The samples *A4*_*1* - *A4*_*3* were analyzed, and the resulting contour plots are shown in Figs. 10, 11, and 12. Each spot on the contour plot represents a single compound, for which a full mass spectrum is available. The x-axis represents the retention times on the *1*st column, indicating the vapor pressure of the analytes. The y-axis shows the *2*nd column retention times, indicating the polarity of the analytes. The spots are labelled with numbers, and homologues series are grouped together marked with arrows. Peak identification was based on mass spectral data from NIST/W9N08 libraries. The mass spectral match factors of the proposed compounds are written in brackets next to the compounds. The following description of the detected spots will assume only the spots occurring in samples *A4*_*1* to *A4*_*3* and not those occuring in the procedural blank (Appendix Fig. 15). The procedural blank was taken by performing a liquid-liquid extraction with water and pure *n*-hexane. After shaking overnight, the organic phase was separated and diluted in a ratio of *1*:*10* and *1*:*5*. The image of the procedural blank is showing spots, which originate from column bleeding, impurities in the solvent or contaminations during the extraction step. So these spots can be automatically excluded in the sample images of the Miller-type experiments. Figure 10 shows the result of the first sample *A4*_*1*+. Fig. 10b shows the blow-up of the marked region of interest depicted in Fig. 10a, illustrating the spots more clearly. Spots numbered 1–3 were proposed to be branched and linear *C11*-*C13* fatty alcohols, whereas the spots 4–6 were assigned to branched *C14*-*C19* alkanes. For the spots 9–10, the libraries suggested mainly *C19*-*C20* alkenes, and for spots 11–13 branched *C16*-*C18* fatty alcohols. Spots 21–27 were proposed as fluorinated fatty acid esters. The fluorine atoms most likely originate from the Teflon discs, which were used to seal the experimental set-up. More polar substances like an alkylated benzoquinone (7) and also phenol (8) were found (Fig. 13). Analyzing sample *A4*_*1*+ (Fig. 10), the most intensive spots are dibutyl phthalate (53), octadecyl vinyl ether (56), and bis(*2*-ethylhexyl)ester hexanedioic acid (57) (Fig. 13). The appearing homologues series from spots 28–34 was proposed to originate from the straight chain alkanes *C16*-*C28*. Spots 35–42 and 45–49 were again fluorinated fatty acid esters in the range from *C24* to *C36*. For spots 50–52, the library proposed longer straight chain alkanes. Due to the fact that electron impact ionization causes strong fragmentation of the analytes, the NIST/W9N08 databases proposed similar alkanes with a high match factor. A clear identification without standards is not possible, and we only classified them. The analysis of the sample *A4*_*2*- (Fig. 11) led to the same spots observed in *A4*_*1*+. Although these analyses were performed only qualitatively, in some cases variations in the spot intensity were visible. Fig. 12 shows the contour plot of the sample *A4*_*3*+. Prior to analysis we injected *50 nmol* of *H*_*2*_ *O*_*2*_ into the running experiment. Spot 59 was only appearing in this sample, which is assigned to *2*,*2*′-Methylenebis(*6*-tert-butyl-*4*-ethylphenol) (Fig. 13) with a match factor of 89, and could act as spin trap. It was possible to identify a lot of branched and unbranched alkanes, alkenes, and fatty alcohols. Also phenols, vinyl ether, and combinations of esters with an acid group were detected. Based on the liquid-liquid-extraction in *n*-hexane/water and the fact that the organic phase was analyzed, almost no compounds containing nitrogen were proposed from the databases, which could be due to the higher solubility of amines, amides, and other nitrogen containing compounds in the hydrophilic phase in conjunction with the properties of the chosen column.

## Discussion

Right after the beginning of the Miller-Urey-type experiment, the broth consists of a gaseous phase and an aqueous phase with an oil-layer on top . We analyzed the gaseous phase by GC/MS, the aqueous phase by LC/MS, NMR, and CARS spectroscopy and the oil phase by GC/MS and GCxGC/MS. We estimated the concentration of several reaction products of the liquid phase by CARS spectroscopy, GC/MS and GCxGC/MS. The molar concentrations of different molecules strongly varied, reaching up to millimolar levels.

NMR spectroscopy performed on samples from identical and different set-ups (Fig. 1), revealed a high variability among the sample composition of various experiments (Figs. 2, 3, 4, and 5), although the starting conditions were as similar as possible. A potential reason for this are sequential reactions where the pool of product molecules determines (likewise catalytically) the next reaction step. Such reaction networks consisting of thousands of substances easily become non-linear, a fortiori as catalysts participate. Non-linearities can drive the system to different compositions despite almost identical initial conditions. This is reminiscent of biological systems (Braun 2015).

NMR spectroscopy of liquid fractions of both types of set-up revealed the presence of alkanes, alkenes, and alkynes as well as aliphatic carbons. Further, NMR spectroscopy exhibited a pronounced signature of *C*-*O* bonds as present in alcohols, acids, and ketones. Besides amines, compounds containing both amine and ketone bonds were detected. Among them amides (especially formamide), urea, and glycine molecules. They are known as important biorelevant precursor molecules (Bada and Lazcano 2003).

NMR spectroscopy also detected cyano compounds (and possibly isocyanate, and azide groups). They are classed as pseudohalogens (Moss et al. 1995) because they are able to chemically act as halogens. Halogens are effective catalysts for many organic reactions.

CARS spectroscopy revealed distinct vibrational features of molecular moieties, depending on the set-up used for sample preparation (Fig. 7). While characteristic spectral signatures from *C*≡*N* triple-bonds and from aliphatic *CH*_*2*_ groups were observed if the broth was prepared with set-up *I*, the absence of both of these signatures and the observation of aromatic ring breathing modes containing *C* and *N* moieties in samples prepared with set-up *II* point to a higher aromaticity in samples from set-up *II*. This dependence of chemical composition on sample preparation was independently confirmed by NMR spectroscopy, performed in parallel on the same set of samples.

Both NMR and CARS spectroscopies identified a lot of nitrogen compounds like amides and *C*≡*N* triple bonds in samples prepared in set-up *I*. The absence of nitriles in samples produced in set-up *II* could be explained by the positioning of the electric discharge. Sparking on the water surface locally increases the concentration of water vapor. The high concentration of oxygen in the area of the electric discharge could increase the formation of oxygen containing compounds compared to nitrogen containing molecules.

The results of NMR and CARS showed high variances in the composition of the reaction mixture dependent on the positioning of the electric discharge. Miller observed considerable differences in the chemical composition of the primordial broth, using an electric spark or a silent discharge, whereas changes of the gas mixture did not affect the chemical variety (Bada and Lazcano 2003).

The oil-like organic layer that formed on top of the water surface in the course of the experiment primarily consisted of branched and linear alkanes, alkenes, fatty alcohols, esters, and ethers, as shown by GC/MS and GCxGC/MS. The number of carbon atoms per molecule varies from *4* up to *28* and above. Benzene rings also represent a significant fraction. Many molecules of the oil phase contained oxygen. Such compounds combine a hydrophobic and a hydrophilic part. They act as tensioactives. A significant volume of foam formed upon shaking, highlighting the presence of tensides. Q-Tof mass spectra of the foam showed PEG derivatives in high intensity compared to all other detected molecules. This indicates the amphiphilic character of the PEG-alkane polymers (Wollrab et al. 2015).

GC/MS of gaseous samples produced in set-up *I* showed a high quantity and important diversity of molecules especially at the beginning of the experiment. The most abundant molecular compounds were benzene rings followed by alkynes, esters, and carbonyls (Table 2). The molecular variety of benzene rings, esters, and carbonyls gradually decreased in the gas phase, while increasing in the liquid medium. It is likely that these compounds increasingly dissolved in the liquid fraction over time. The variety of alkenes, and hydroxyls diminished with time in the gas phase, whereas they remained at a constant concentration in the liquid phase. Alkynes, alkanes and ethers constituted a permanent fraction of the gaseous phase (Table 2). Molecules containing nitrogen groups like nitriles and amines constituted an exception since they appeared and disappeared over the experimental time course (Table 2). Among the detected molecular pool we found highly reactive and marginally stable functional groups like nitriles, epoxides, and a lot of diketones or polyphenols (Figs. 8 and 9). The ketone groups in diketones and polyphenols were often arranged symmetrically. In this configuration they are able to act as radical traps. Prominent examples are quinones which play an important role in biology (Creber et al. 1982). They can be produced by auto-oxidation of polyphenols under high pH-values (Gutierrez 2000; Nair et al. 2004). Polyphenols are well-known antioxidants in phytochemistry (Scalbert et al. 2005). After formation, quinones are able to reduce to semiquinones and release superoxides (Gutierrez 2000; Nair et al. 2004). Due to the highly alkaline conditions of the reaction mixture (pH-value between 9 and 11), diketones are able to attract dipolar and positively charged molecules and act as oxidizers.

Comparing the different techniques, first of all we note that the results from GC/MS and GCxGC/MS were very similar. They revealed not only carbon chains of different length and benzene rings, but also complex molecules. As long as they primarily describe the oil-like phase, they can hardly be compared to the results of NMR and CARS, which were only able to analyze the water-based fraction due to the small quantity of extracted hydrophobic compounds. The results from NMR and CARS spectroscopies partially vary, but they are not contradictory. The concentration of molecular compounds in the primordial broth is generally very low, not exceeding the micromolar scale. Consequently, the chemical specification is performed at the limit of sensitivity of the method used for analysis. Another possible explanation for observed differences is that the large molecular variety caused overlap of several signals that made sometimes a clear identification of molecular compounds impossible. Under these conditions, the chemical analyses by NMR and CARS spectroscopies can complement each other.

We previously showed that different polymers arise in the reaction mixture. Mass spectrometry revealed polyethylene glycol (PEG) derivatives, while NMR spectroscopy revealed polymers containing a nitrogen-carbon backbone (Wollrab et al. 2015).

In highly alkaline solutions, *HCN* can produce the tetramer diaminomalonitrile (DAMN), and an interconnected ladder polymer (Völker 1960; Matthews and Minard 2006). In comparison, the production of the here observed nitrogen-based polymer is not that preferential (Wollrab et al. 2015). In alkaline solutions, after the reaction of *HCN* molecules to the trimer aminomalonitrile, polyaddition of the trimer forms polyaminomalonitrile, which cumulatively reacts with *HCN* to heteropolyamidines. In water, the trimers react to heteropolypeptides by releasing NH_3_ and CO_2_ (Matthews and Moser 1966, 1967). The heteropolyamidines and heteropolypeptides are similar to the nitrogen-based polymer in our reaction mixture. We assume related formation steps which are possibly directed by catalysts. As the polymer consists of a nitrogen-carbon backbone, it is also located in the oil/water interface. There, it can possibly participate as a template in the formation process of the PEG polymers.

The detected PEG derivatives are bound to carbon chains of approximately *12* carbon atoms (Wollrab et al. 2015). These tensides are soluble in the aqueous phase as well as in the oil phase. PEG is a well-known phase-transfer catalyst, capable of transporting positively charged molecules from the aqueous phase into the oil phase and vice versa (Kim et al. 2003b; Totten and Clinton 1988).

Neither NMR spectroscopy nor GC/MS and GCxGC/MS, showed strong distinctions in the chemical composition between samples containing PEG or not in the same set-up. We conclude that only a few different molecules participate in the formation and degradation of the polymers. They are hard to identify among the large molecular pool.

The PEG polymers are not stable within the broth (Wollrab et al. 2015). It appeared that the polymers were much more likely to appear in set-up *II* compared to set-up *I*. In set-up *II*, PEG was detected in over *90*% of the samples. In this set-up more oxygen radicals were produced, because of directly sparking onto the aqueous phase. We observed that after injection of *H*_*2*_ *O*_*2*_, PEG polymers formed. Exemplarily, we listed samples *A4*_*2*- and *A4*_*3*+ (Table 1). The purpose of adding H_2_O_2_ was to test for the influence of a radical former on the reaction mixture. In the experiment radicals form during the sparking and they exhibit a short life time. Furthermore, GCxGC showed *2*,*2*′-Methylenebis(*6*-tert-butyl-*4*-ethylphenol) which appeared in the hydrophobic phase in *A4*_*3*+. *2*,*2*′-Methylenebis(*6*- tert-butyl-*4*-ethylphenol) is a polyphenol (an antioxidant (Scalbert et al. 2005)) and strongly reminds of a reduced quinone. If a quinone or diketone is reduced, a superoxide gets released that oxidizes other molecules. We understand that the addition of *H*_*2*_ *O*_*2*_ shifted the system into a more oxidizing state where PEG derivatives formed.

Furthermore in set-up *II*, NMR and CARS spectroscopies did not detect clear signals for cyanide compounds in the aqueous phase. Since the PEG polymers were much more stable in this set-up, this points towards cyanides promoting the degradation of PEG polymers. Nitriles are strong reducing agents. Byproducts of reduction reactions induced by nitriles are for example amides that were highly present in the NMR spectra.

The amphiphilic PEG derivatives have to be located in the interface of the aqueous medium and the oil-layer. This points towards interfacial catalysis. Under alkaline conditions and with respect to the presence of oxygen radicals, it is very likely that nitrogen oxides are steadily present in the aqueous phase. Partially, they may gain a positive charge from dissolved ammonium cations in the water to form positively charged quaternary ammonia such as nitro compounds (Fig. 9). Charge bearing hydrophobic molecules will accumulate and, at the interface, attract *OH*- ions or *OH*∙ radicals. These aggressive oxygen components degrade the hydrophobic layer. As product molecules, PEG derivatives offer an energetically beneficial structure. In agreement with this idea, the relatively short hydrophobic tail is compatible with marginal stability in the interface (Dose and Rauchfuss 1975).

In conclusion, in the Miller-Urey-type experiment a spontaneously forming oil/water interface offers the possibility of interfacial catalysis. The interface is likely to lead to the production of oxidized amphiphiles, among them PEG derivatives, through oxidizing and radical containing aqueous phase.

In future studies, CARS microspectroscopy could offer a powerful tool for the non-invasive, in-situ, chemical mapping (Volkmer 2010) of complex Miller-Urey-type samples.

## Electronic supplementary material

Below is the link to the electronic supplementary material.

ESM 1 (PDF 1819 kb)
