# Supplementary material for: Chemical Analysis of a “Miller-Type” Complex Prebiotic Broth: Part II: Gas, Oil, Water and the Oil/Water-Interface
Source: Orig Life Evol Biosph. 2016 Nov 28;47(4):381–403. doi: 10.1007/s11084-016-9528-8 (PMC5705758; doi:10.1007/s11084-016-9528-8)
Supplement: Supplementary file 1 — (PDF 1819 kb) [file 11084_2016_9528_MOESM1_ESM.pdf]

Name	Molecular formula	Structure	Probability [%]	Delay time [min]	Relative height/ area [% / %]
Methyl alcohol	CH_4O	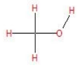	43.5	0.28	80.63 / n.a.
Methane	CH_4	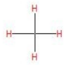	21.1		
3-Methyl-1,2-diazirine	$C_2H_4N_2$	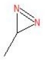	54.6	0.28	100 / n.a.
Acetylene	C_2H_2		96.5	0.30	88.47 / 100
1-Buten-3-yne	C_4H_4		95.2	0.31	11.91 / n.a.
1,3-Cyclopentadiene, 1-methyl	C_6H_8	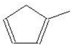	35.3	0.33	5.00 / 2.71
Benzene	C_6H_6	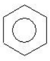	77.3	0.43	7.08 / 26.08
1,3-Butadiyne	C_4H_2		98.7	0.45	8.28 / n.a.
Hepta-4,6-diyn-2-ol	C_7H_8O	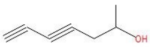	67.8	0.48	5.22 / n.a.
Toluene	C_7H_8	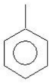	47.7	0.59	4.42 / 38.89
Water	H_2O	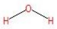	92.1	0.70	18.76 / n.a.
Hepta-4,6-diyn-2-ol	C_7H_8O	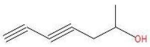	60.2	0.71	12.34 / 39.62
Ethylbenzene	C_8H_{10}	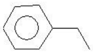	61.0	0.85	4.29 / 3.91
2,4-Hexadiyne	C_6H_6		43.6	0.91	4.04 / 4.49
1,5-Hexadien-3-yne	C_6H_6	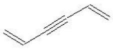	21.2		
Bicyclo[4.1.0]octa-1,3,5-triene-7,8-dione	$C_8H_4O_2$	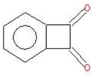	48.8	1.07	3.14 / 27.35
Oxiranecarboxamide, 3-phenyl-	$C_9H_9NO_2$	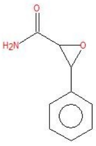	54.5	1.10	3.85 / n.a.

Bicyclo[4.1.0]hepta-1,3,5-triene	C_7H_6	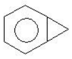	88.8	1.32	3.19 / 7.23
Benzene, 1,2,3-trimethyl-	C_9H_{12}	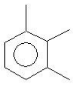	12.4	1.37	3.07 / 2.94
Benzene, (1-methylethyl)-	C_9H_{12}	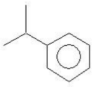	8.27		
1-Propoxypropan-2-yl benzoate	$C_{13}H_{18}O_3$	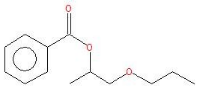	8.27		
Styrene	C_8H_8	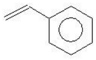	30.6	1.61	3.44 / 9.49
Bicyclo[4.1.0]octa-1,3,5-triene	C_8H_8	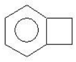	28.3		
1,3,5,7-Cyclooctatetraene	C_8H_8	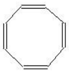	26.1		
Hexa-1,3,5-triyne	C_6H_2	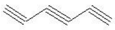	96.7	2.56	2.66 / 3.53
Phenylethyne	C_8H_6	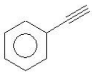	89.4	2.82	2.75 / 7.02
1H-Indene, 1-chloro-2,3-dihydro-	C_9H_9Cl	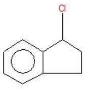	29.1	4.21	2.04 / 2.86
Indene	C_9H_8	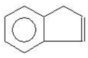	26.9		
Benzene, 1-propynyl	C_9H_8	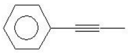	24.7	4.54	1.72 / 6.64
2-Methylphenylacetylene	C_9H_8	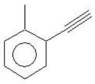	23.8		
2-Thiazolidinecarboxylic acid	$C_4H_7NO_2S$	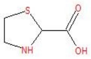	32.9	4.68	2.14 / 2.86

<table>
<tr><td>Benzene, 1-ethynyl-4-methyl-</td><td>C_9H_8</td><td>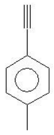</td><td>50.0</td><td>5.04</td><td>1.44 / 2.08</td></tr>
<tr><td>Divinyl(N-methylcarbamoyloxy methyl)silane</td><td>$C_8H_{15}NO_2Si$</td><td>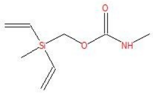</td><td>34.6</td><td>5.70</td><td>1.58 / 3.22</td></tr>
<tr><td>2-Methylindene</td><td>$C_{10}H_{10}$</td><td>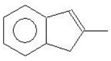</td><td>21.5</td><td rowspan="3">5.76</td><td rowspan="3">1.86 / 2.36</td></tr>
<tr><td>1,4-Methanonaphthalene-9-one, 1,2,3,4-tetrahydro-</td><td>$C_{11}H_{10}O$</td><td>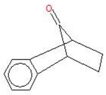</td><td>16.5</td></tr>
<tr><td>1H-Indene, 3-methyl-</td><td>$C_{10}H_{10}$</td><td>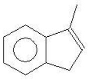</td><td>10.6</td></tr>
<tr><td>d-Proline, N-methoxycarbonyl-, nonyl ester</td><td>$C_{16}H_{29}NO_4$</td><td>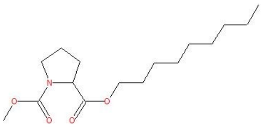</td><td>7.44</td><td rowspan="3">7.79</td><td rowspan="3">1.41 / 4.75</td></tr>
<tr><td>d-Proline, N-methoxycarbonyl-, dodecyl ester</td><td>$C_{19}H_{35}NO_4$</td><td>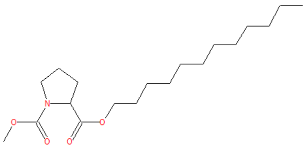</td><td>5.84</td></tr>
<tr><td>d-Proline, N-methoxycarbonyl-, octadecyl ester</td><td>$C_{25}H_{47}NO_4$</td><td>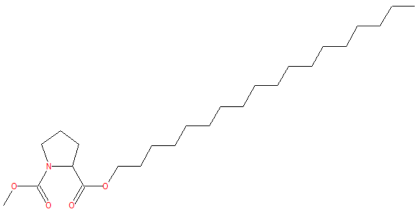</td><td>4.94</td></tr>
<tr><td>4a,8a-(Methaniminomethano)naphthalene-9,11-dione, 10-phenyl-</td><td>$C_{18}H_{13}NO_2$</td><td>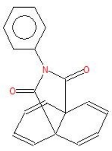</td><td>46.90</td><td>7.87</td><td>1.42 / 5.30</td></tr>
</table>

Acetamide, 2-(1-piperidyl)-N-(6-chloropyrazin-2-yl)-	$C_{11}H_{15}ClN_4O$	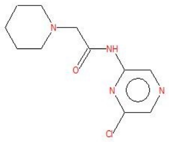	29.3	8.08	1.69 / 5.89
Naphthalene	$C_{10}H_8$	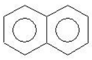	49.5	8.32	1.10 / n.a.
Azulene	$C_{10}H_8$	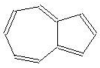	37.9		
1,4-Diethynylbenzene	$C_{10}H_6$	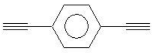	47.9	10.50	1.23 / 3.96
Carbonic acid, phenyl undec-10-enyl ester	$C_{18}H_{26}O_3$	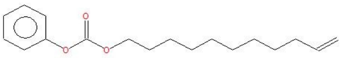	22.3	15.36	0.69 / 3.93
Carbonic acid, pentadecyl phenyl ester	$C_{22}H_{36}O_3$	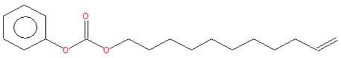	16.6		
Acenaphthylene	$C_{12}H_8$	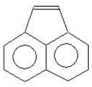	64.5	15.45	0.84 / 4.94
Caprolactam	$C_6H_{11}NO$	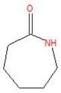	78.0	16.00	0.60 / n.a.
Vinyl 2-ethylhexanoate	$C_{10}H_{18}O_2$	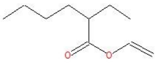	30.4	18.37	0.75 / 3.38
1,2(4H9)-Oxazine-3-ol, 5,6-dihydro-6-oxo, benzoate	$C_{11}H_9NO_4$	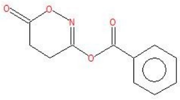	19.1	19.62	0.63 / 1.48
9H-Fluorene, 9-methylene	$C_{14}H_{10}$	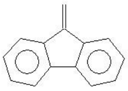	28.9	22.33	0.70 / 4.00
Phenanthrene	$C_{14}H_{10}$	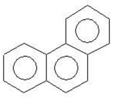	25.6		

<table>
<tr><td>Phthalic acid, 4-cyanophenyl heptyl ester</td><td>$C_{22}H_{23}NO_4$</td><td>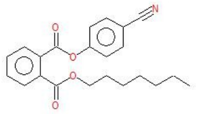</td><td>12.4</td><td rowspan="2">23.24</td><td rowspan="2">0.48 / n.a.</td></tr>
<tr><td>Phthalic acid, cyclobutyl tridecyl ester</td><td>$C_{25}H_{38}O_4$</td><td>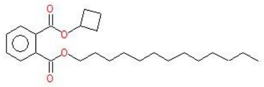</td><td>8.26</td></tr>
<tr><td>Resorcinol</td><td>$C_6H_6O_2$</td><td>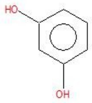</td><td>18.9</td><td rowspan="2">27.51</td><td rowspan="2">0.68 / 3.53</td></tr>
<tr><td>Hydroquinone</td><td>$C_6H_6O_2$</td><td>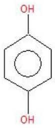</td><td>18.9</td></tr>
</table>

Table 1: Detected molecules from $A3\_1_{gas}$ as obtained using the NIST database as a reference. For low-percentage matches several possible molecules are listed. The highest peak, or the largest peak area in the chromatogram are used as references. Heights and areas of other peaks from other substances are given as a percentage relative to the respective reference and taken as their relative abundances. Not available (n.a.) area means that the area could not be determined due to asymmetric or overlapping peaks. Note that both estimates (height vs. area) can differ more or less, depending on the precise shape of a peak. Molecules containing elements other than C, H, N and O may represent mismatches in the database, or a contamination from the cleaning solution (*Deconex*, Borer).

Name	Molecular formula	Structure	Probability [%]	Delay time [min]	Relative height/ area [% / %]
3-Methyl-1,2-diazirine	$C_2H_4N_2$	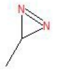	66.1	0.30	100 / 0.28
1-Buten-3-yne	C_4H_4		95.2	0.31	99.55 / 100
Ammonia	H_3N	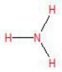	98.0	0.35	12.68 / 10.62
2-Cyanosuccinonitrile	$C_5H_3N_3$	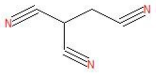	59.4	0.44	5.10 / 0.62
1,3-Butadiyne	C_4H_2		91.2	0.46	9.11 / 6.34
Sulfur dioxide	O_2S	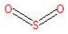	69.0	0.49	6.46 / 1.81
Toluene	C_7H_8	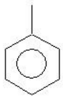	50.2	0.61	2.59 / n.a.
Water	H_2O	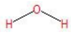	94.7	0.71	13.06 / n.a.
Hepta-4,6-diyn-2-ol	C_7H_8O	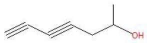	46.5	0.72	10.81 / n.a.
Ethylbenzene	C_8H_{10}	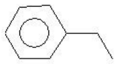	43.1	0.88	2.12 / 0.27
Benzene, 1,3-dimethyl-	C_8H_{10}	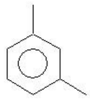	27.0		
Bicyclo[4.2.0]octa-1,3,5-triene-7,8-dione	$C_8H_4O_2$	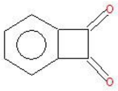	49.2	1.08	2.00 / 0.72
4,6-Octadiyn-3-one, 2-methyl-	$C_9H_{10}O$	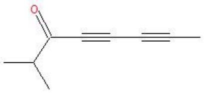	77.0	1.18	1.90 / 0.36
Bicyclo[4.1.0]hepta-1,3,5-triene	C_7H_6	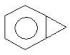	83.5	1.33	1.46 / n.a.
Acetic acid, sodium salt	$C_2H_3NaO_2$	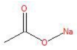	90.8	1.40	1.52 / n.a.
1,3,5,7-Cyclooctatetraene	C_8H_8	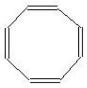	34.6		

Styrene	C_8H_8	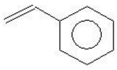	26.5	1.68	1.50 / 0.29
Bicyclo[4.2.0]octa-1,3,5-triene	C_8H_8	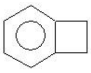	24.5		
Bicyclo[4.1.0]hepta-1,3,5-triene	C_7H_6	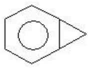	56.5	2.32	1.41 / 0.46
Hexa-1,3,5-triyne	C_6H_2	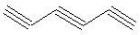	96.8	2.58	1.22 / 0.51
3-Phenyl-1-propanol, acetate	$C_{11}H_{14}O_2$	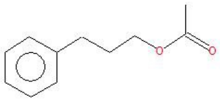	22.5	2.66	1.29 / 0.30
Indane	C_9H_{10}	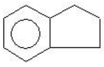	14.6		
Propanoic acid, 2-methyl-, 3-phenylpropyl ester	$C_{13}H_{18}O_2$	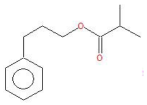	11.2		
Phenylethyne	C_8H_6	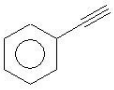	88.8	2.85	1.26 / n.a.
Indene	C_9H_8	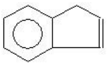	23.1	4.26	1.06 / n.a.
Benzene, 1-propynyl-	C_9H_8	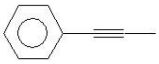	16.3		
Benzene, 1-ethynyl-4-methyl-	C_9H_8	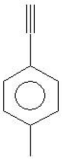	13.8		
2-Thiazolidinecarboxylic acid	$C_4H_7NO_2S$	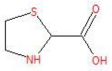	35.3	4.69	1.06 / 0.48
Benzene, 1-ethynyl-4-methyl-	C_9H_8	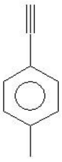	83.9	5.02	1.14 / n.a.

Thiophene, 2-(methylthio)-	$C_5H_6S_2$	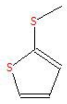	48.5	5.75	0.80 / n.a.
1H-Phosphole, 2,3-dihydro-1,4-dimethyl-, 1-oxide	$C_6H_{11}OP$	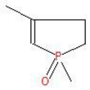	32.3		
l-Norvaline, N-methoxycarbonyl-, heptyl ester	$C_{14}H_{27}NO_4$	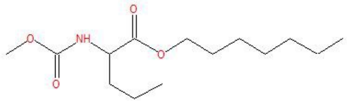	26.2	5.85	0.92 / n.a.
l-Norvaline, N-methoxycarbonyl-, octyl ester	$C_{15}H_{29}NO_4$	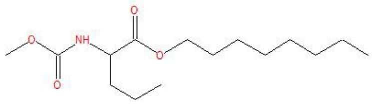	16.9		
l-Norvaline, N-methoxycarbonyl-, pentyl ester	$C_{12}H_{23}NO_4$	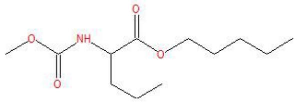	16.3		
Benzonitrile	C_7H_5N	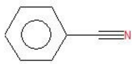	73.2	6.29	0.86 / 0.52
2,4,5-Trihydroxypyrimidine	$C_4H_4N_2O_3$	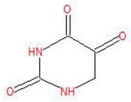	25.6	6.74	0.89 / 0.39
1-[2-Pyridyl]-2,2-dimethyl-2-morpholino ethanol	$C_{13}H_{20}N_2O_2$	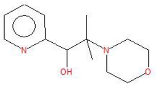	20.1		
1,3,4-Oxadiazole-2(3H)-thione, 3-(2,6-dimethylmorpholinomethyl)-5-(2-methylphenyl)-	$C_{16}H_{21}N_3O_2S$	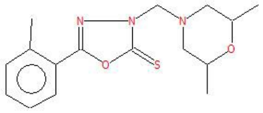	19.3		
Etidocaine	$C_{17}H_{28}N_2O$	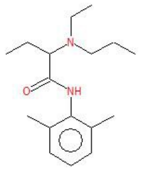	41.0	7.83	0.65 / n.a.
Naphthalene	$C_{10}H_8$	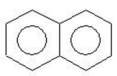	58.0	8.30	0.52 / n.a.

l-Alanine, n-propargyloxycarbonyl-, ethyl ester	$C_9H_{13}NO_4$	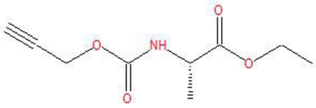	31.4	8.87	0.51 / n.a.
Naphthalene, 2-methyl-	$C_{11}H_{10}$	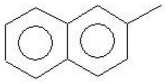	76.5	10.15	0.58 / 0.23
l-Alanine, n-propargyloxycarbonyl-, ethyl ester	$C_9H_{13}NO_4$	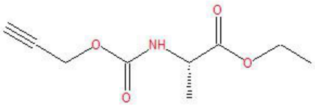	21.1	10.48	0.57 / 0.30
D-Alanine, N-propargyloxycarbonyl-, propargyl ester	$C_{10}H_{11}NO_4$	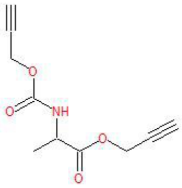	14.9		
Naphthalene, 2-methyl-	$C_{11}H_{10}$	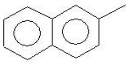	39.5	10.72	0.41 / n.a.
Naphthalene, 1-methyl-	$C_{11}H_{10}$	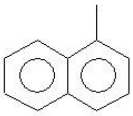	20.4		
Acenaphthylene	$C_{12}H_8$	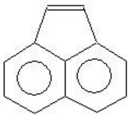	48.1	15.46	0.33 / n.a.
Biphenylene	$C_{12}H_8$	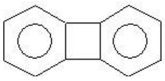	31.1		
Benzo[f]isoindol-1,3(1H,3H)-dione, 3a,4,9,9a-tetrahydro-4,9-O-benzeno-2-hexyl-	$C_{24}H_{25}NO_2$	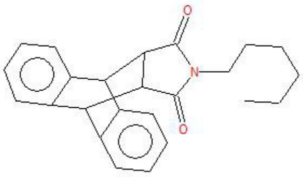	22.3	22.59	0.29 / n.a.

Table 2: Detected molecules from $A3\_2_{gas}$ as obtained using the NIST database as a reference. For low-percentage matches several possible molecules are listed. The highest peak, or the largest peak area in the chromatogram are used as references. Heights and areas of other peaks from other substances are given as a percentage relative to the respective reference and taken as their relative abundances. Not available (n.a.) area means that the area could not be determined due to asymmetric or overlapping peaks. Note that both estimates (height vs. area) can differ more or less, depending on the precise shape of a peak. Molecules containing elements other than C, H, N and O may represent mismatches in the database, or a contamination from the cleaning solution ($Deconex$, Borer).

Name	Molecular formula	Structure	Probability [%]	Delay time [min]	Relative height/ area [% / %]
3-Methyl-1,2-diazirine	$C_2H_4N_2$	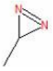	60.8	0.28	100 / 100
1-Buten-3-yne	C_4H_4		96.7	0.30	24.48 / 9.30
Ammonia	H_3N	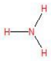	98.5	0.34	12.68 / 2.73
Benzene	C_6H_6	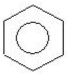	69.6	0.44	10.81 / 3.91
1,3-Butadiyne	C_4H_2		97.9	0.46	12.79 / 6.22
Hepta-4,6-diyn-2-ol	C_7H_8O	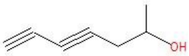	72.4	0.51	9.19 / 0.62
Methyl isocyanide	C_2H_3N		42.0	0.56	7.65 / 1.99
Acetonitrile	C_2H_3N		33.0		
Toluene	C_7H_8	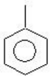	45.1	0.58	8.62 / 2.29
Water	H_2O	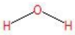	98.8	0.69	19.92 / 32.18
Hepta-4,6-diyn-2-ol	C_7H_8O	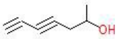	48.1	0.71	16.54 / 7.62
Ethylbenzene	C_8H_{10}	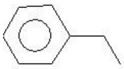	29.7	0.85	7.01 / 1.35
Benzene, 1,3-dimethyl-	C_8H_{10}	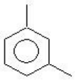	25.1		
Bicyclo[4.2.0]octa-1,3,5-triene-7,8-dione	$C_8H_4O_2$	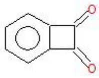	44.8	1.05	6.53 / 0.68
4,6-Octadiyn-3-one, 2-methyl-	$C_9H_{10}O$	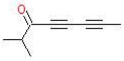	87.3	1.14	5.48 / 2.27
Bicyclo[4.1.0]hepta-1,3,5-triene	C_7H_6	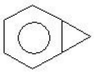	45.3	1.29	5.56 / 0.18
Styrene	C_8H_8	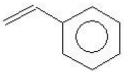	31.0	1.64	5.17 / 0.85
Bicyclo[4.2.0]octa-1,3,5-triene	C_8H_8	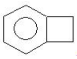	23.7		
Hexa-1,3,5-triyne	C_6H_2	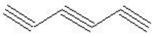	92.1	2.54	4.66 / 0.71

Phenylethyne	C_8H_6	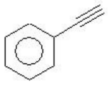	82.3	2.83	4.58 / 0.43
Benzene, 1-ethynyl-4-methyl-	C_9H_8	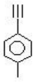	33.7	4.25	3.63 / 0.90
Benzene, 1-propynyl-	C_9H_8	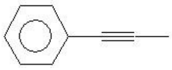	21.8		
d-Proline, N-methoxycarbonyl-, octadecyl ester	$C_{25}H_{47}NO_4$	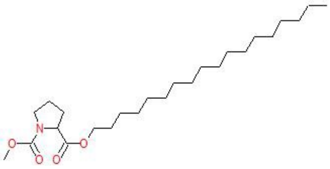	9.55	8.38	2.50 / 0.90
d-Proline, N-methoxycarbonyl-, tetradecyl ester	$C_{21}H_{39}NO_4$	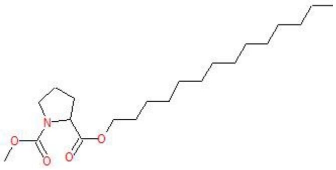	5.98		
d-Proline, N-methoxycarbonyl-, hexadecyl ester	$C_{23}H_{43}NO_4$	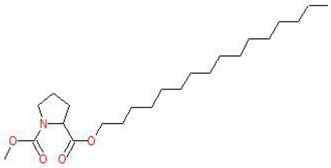	5.98		
1,3-Benzenediol, o-methoxycarbonyl-o'-(2-methoxybenzoyl)-	$C_{16}H_{14}O_6$	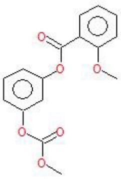	14.9	9.50	1.53 / n.a.
1,2-Benzenediol, o-(4-methoxybenzoyl)-o'-(2,2,3,3,4,4,4-heptafluorobutyryl)-	$C_{18}H_{11}F_7O_5$	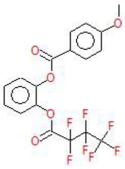	10.2		
1,2-Benzisothiazole	C_7H_5NS	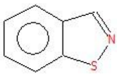	44.2		

1H-Pyrazolo[3,4-d]pyrimidin-4-amine	$C_5H_5N_5$	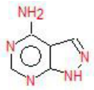	37.4	11.99	1.32 / n.a.
2-Ethyl-hexoic acid	$C_8H_{16}O_2$	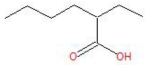	58.4	12.56	1.61 / 0.37
3-Methylbutyl 2-ethylhexanoate	$C_{13}H_{26}O_2$	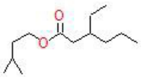	18.4	13.56	1.10 / n.a.
Hexanoic acid, 2-ethyl-, 1,1-dimethylethyl ester	$C_{12}H_{24}O_2$	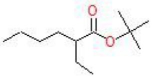	16.2		
Iodoacetylene	C_2HI		84.7	15.46	1.31 / 0.60
Carbon disulfide	CS_2		63.0	20.11	0.93 / n.a.

Table 3: Detected molecules from $A3\_3_{gas}$ as obtained using the NIST database as a reference. For low-percentage matches several possible molecules are listed. The highest peak, or the largest peak area in the chromatogram are used as references. Heights and areas of other peaks from other substances are given as a percentage relative to the respective reference and taken as their relative abundances. Not available (n.a.) area means that the area could not be determined due to asymmetric or overlapping peaks. Note that both estimates (height vs. area) can differ more or less, depending on the precise shape of a peak. Molecules containing elements other than C, H, N and O may represent mismatches in the database, or a contamination from the cleaning solution (*Deconex*, Borer). Fluorine may originate from the teflon used for sealing.

Name	Molecular formula	Structure	Probability [%]	Delay time [min]	Relative height/ area [% / %]	Concentration [(± 1.026) mol/l]
Pentane	C_5H_{12}	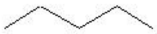	80.8	0.12	14.97 / 8.76	0.401
Ethyl ether	$C_4H_{10}O$	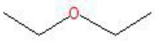	87.7	0.13	13.68 / 7.83	0.389
Cyclohexane	C_6H_{12}	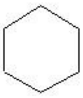	76.0	0.14	9.48 / 5.75	0.232
Pentane	C_5H_{12}	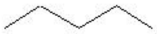	53.5	0.29	100 / 100	3.031
Ethyl ether	$C_4H_{10}O$	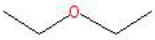	73.5	0.30	62.80 / 62.79	2.614
Cyclohexane	C_6H_{12}	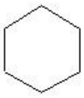	74.4	0.32	54.29 / 73.32	**3.090**
4-Hydroxy-5-methyl-3-phenyl-δ(2)-1,2,4-oxadiazoline	$C_9H_{10}N_2O_2$	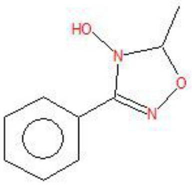	37.0	22.60	0.28 / 0.35	0.007

Table 4: Detected molecules from $A3\_1_{liquid}$ as obtained using the NIST database as a reference. For low-percentage matches several possible molecules are listed. The highest peak, or the largest peak area in the chromatogram are used as references. Heights and areas of other peaks from other substances are given as a percentage relative to the respective reference and taken as their relative abundances. Not available (n.a.) area means that the area could not be determined due to asymmetric or overlapping peaks. Note that both estimates (height vs. area) can differ more or less, depending on the precise shape of a peak. For estimating the molar concentration, we compared the areas and used the known solvent concentration as reference (printed in bold).

Name	Molecular formula	Structure	Probability [%]	Delay time [min]	Relative height/ area [% / %]	Concentration [(± 1.911) mol/l]
1-Propanol, 2-methyl-	$C_4H_{10}O$	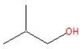	62.2	0.23	19.45 / 33.27	2.073
Cyclohexane	C_6H_{12}	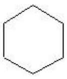	78.9	0.25	12.04 / 13.06	1.032
Butane, 2-methyl-	C_5H_{12}	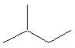	36.1	0.28	100 / 87.44	3.162
Pentane	C_5H_{12}	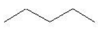	34.7			3.039
Ethane, 1,2-diethoxy-	$C_6H_{14}O_2$	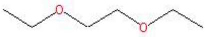	52.5	0.28	83.33 / 34.71	1.825
Ethyl ether	$C_4H_{10}O$	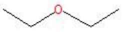	40.3			1.401
2H-Pyran, 3,4-dihydro	C_5H_8O	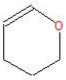	47.3	0.30	71.30 / 100	4.738
Cyclohexane	C_6H_{12}	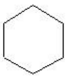	35.4	0.32	32.68 / 87.14	**3.090**
1,2,4-Triazol-3-amine, 5-(3-nitrophenyl)-	$C_8H_7N_5O_2$	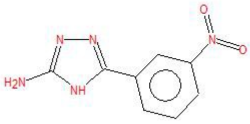	20.1	11.78	0.33 / n.a.	n.a.
p-Benzoquinone, 2,5-bis(1,1,3,3-tetramethylbutyl)-	$C_{22}H_{36}O_2$	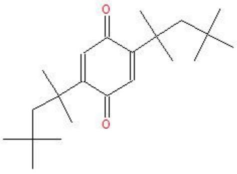	17.7			n.a.
2,6-Bis(1,1-dimethylethyl)-4-methyl-4-isopropylcyclohexa-2,5-dien-1-one	$C_{18}H_{30}O$	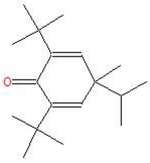	12.9			n.a.
(E)-tert-Butylsulfinyl-2-phenylethene	$C_{12}H_{16}OS$	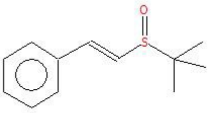	50.1	15.43	0.26 / n.a.	n.a.

1,2,4,5-Benzenetetracarbonitrile	$C_{10}H_2N_4$	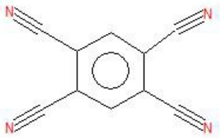	84.7	22.57	0.16 / n.a.	n.a.

Table 5: Detected molecules from $A3\_2_{liquid}$ as obtained using the NIST database as a reference. For low-percentage matches several possible molecules are listed. The highest peak, or the largest peak area in the chromatogram are used as references. Heights and areas of other peaks from other substances are given as a percentage relative to the respective reference and taken as their relative abundances. Not available (n.a.) area means that the area could not be determined due to asymmetric or overlapping peaks. Note that both estimates (height vs. area) can differ more or less, depending on the precise shape of a peak. For estimating the molar concentration, we compared the areas and used the known solvent concentration as reference (printed in bold). Molecules containing elements other than C, H, N and O may represent mismatches in the database, or a contamination from the cleaning solution (*Deconex*, Borer).

Name	Molecular formula	Structure	Probability [%]	Delay time [min]	Relative height/ area [% / %]	Concentration [(± 1.980) mol/l]
1-Propanol, 2-methyl-	$C_4H_{10}O$	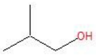	63.5	0.23	4.03 / 2.36	0.240
Cyclohexane	C_6H_{12}	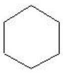	80.7	0.25	4.14 / 0.97	0.126
Pentane	C_5H_{12}	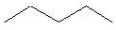	29.2	0.28	100 / 68.64	3.212
2-Oxetanone, 4-methyl-	$C_4H_6O_2$	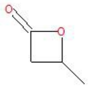	20.6			2.266
Acetic acid, (2-ethoxy)ethoxy-, methyl ester	$C_7H_{14}O_4$	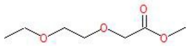	38.6	0.28	85.19 / 36.79	2.276
Allyl isocyanate	C_4H_5NO	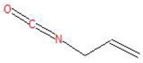	72.1	0.30	70.83 / 100	11.553
Cyclohexane	C_6H_{12}	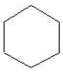	60.5	0.31	50.00 / 31.88	**3.090**
Carbon monoxide	CO		74.2	0.57	1.78 / 0.24	0.028
Water	H_2O	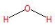	82.9	0.67	2.70 / 3.83	0.509
Di-tert-butyl peroxide	$C_8H_{18}O_2$	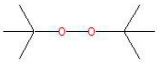	58.9	3.38	0.81 / 0.16	0.014
Oxalic acid, butyl propyl ester	$C_9H_{16}O_4$	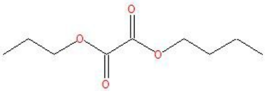	17.6	9.73	0.42 / 0.16	0.005
di-tert-Butyl dicarbonate	$C_{10}H_{18}O_5$	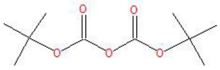	10.1			0.003
Oxalic acid, isobutyl propyl ester	$C_9H_{16}O_4$	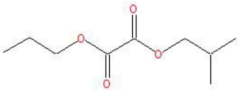	7.95			0.002
1,2-Benzenediol, o-(4-methoxybenzoyl)-o'-(2,2,3,3,4,4,4-heptafluorobutyryl)-	$C_{18}H_{11}F_7O_5$	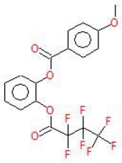	8.07			0.002

1,2-Benzenediol, o-(2-methoxybenzoyl)-o'-(propargyloxycarbonyl)-	$C_{18}H_{14}O_6$	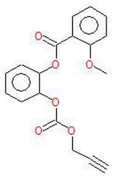	6.81	10.22	0.40 / 0.17	0.002
1,3-Benzenediol, o-(2-methoxybenzoyl)-o'-propargyloxycarbonyl-	$C_{18}H_{14}O_6$	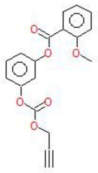	6.28			0.002
Isophthalic acid, butyl 2-isopropylphenyl ester	$C_{21}H_{24}O_4$	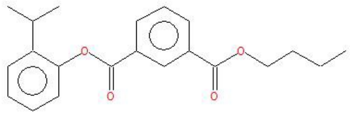	23.7	11.46	0.33 / 0.18	0.007
Terephthalic acid, isobutyl 2-isopropylphenyl ester	$C_{21}H_{24}O_4$	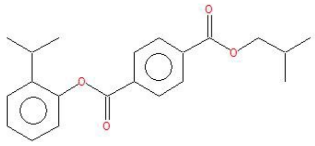	9.38			0.003
Benzothiazole	C_7H_5NS	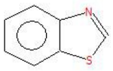	34.0	11.87	0.35 / 0.27	0.015
1H-Pyrazolo[3,4-d]pyrimidin-4-amine	$C_5H_5N_5$	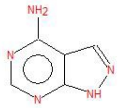	21.3			0.009
Benzothiazole	C_7H_5NS	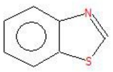	48.7	11.97	0.23 / n.a.	n.a.
1,2-Benzisothiazole	C_7H_5NS	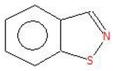	26.6			n.a.
2-Ethyl-hexoic acid	$C_8H_{16}O_2$	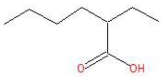	73.2	12.53	0.27 / n.a.	n.a.
3-Methylbutyl 2-ethylhexanoate	$C_{13}H_{26}O_2$	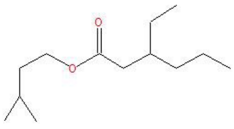	30.2	13.52	0.36 / n.a.	n.a.

Hexanoic acid, 2-ethyl-, 1,1-dimethylethyl ester	$C_{12}H_{24}O_2$	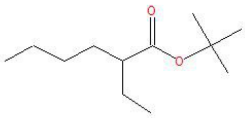	18.3			n.a.

Table 6: Detected Molecules from $A3\_3_{liquid}$ as obtained using the NIST database as a reference. For low-percentage matches several possible molecules are listed. The highest peak, or the largest peak area in the chromatogram are used as references. Heights and areas of other peaks from other substances are given as a percentage relative to the respective reference and taken as their relative abundances. Not available (n.a.) area means that the area could not be determined due to asymmetric or overlapping peaks. Note that both estimates (height vs. area) can differ more or less, depending on the precise shape of a peak. For estimating the molar concentration, we compared the areas and used the known solvent concentration as reference (printed in bold). Molecules containing elements other than C, H, N and O may represent mismatches in the database, or a contamination from the cleaning solution (*Deconex*, Borer). Fluorine may originate from the teflon used for sealing.

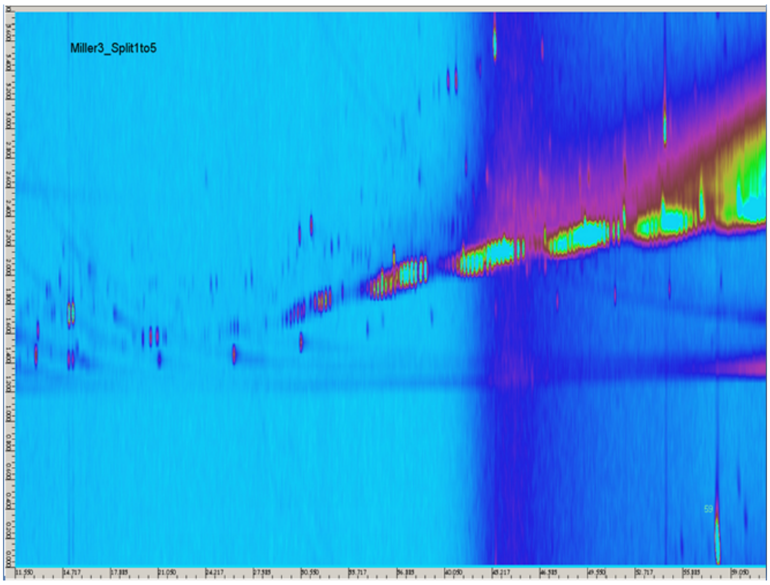

Fig. 1: Contour plot of the procedural blank.
